# Septo-dentate gyrus cholinergic circuits modulate function and morphogenesis of adult neural stem cells through granule cell intermediaries

**DOI:** 10.1073/pnas.2405117121

**Published:** 2024-09-23

**Authors:** Ze-Ka Chen, Luis Quintanilla, Yijing Su, Ryan N. Sheehy, Jeremy M. Simon, Yan-Jia Luo, Ya-Dong Li, Zhe Chen, Brent Asrican, Dalton S. Tart, W. Todd Farmer, Guo-Li Ming, Hongjun Song, Juan Song

**Affiliations:** ^a^Department of Pharmacology, University of North Carolina at Chapel Hill, Chapel Hill, NC 27599; ^b^Neuroscience Center, University of North Carolina at Chapel Hill, Chapel Hill, NC 27599; ^c^Neuroscience Curriculum, University of North Carolina at Chapel Hill, Chapel Hill, NC 27599; ^d^Department of Neuroscience and Mahoney Institute for Neurosciences, Perelman School of Medicine, University of Pennsylvania, Philadelphia, PA 19104; ^e^Department of Oral Medicine, School of Dental Medicine, University of Pennsylvania, Philadelphia, PA 19104; ^f^Pharmacology Curriculum, University of North Carolina at Chapel Hill, Chapel Hill, NC 27599; ^g^Department of Genetics, University of North Carolina at Chapel Hill, Chapel Hill, NC 27599; ^h^Carolina Institute for Developmental Disabilities, University of North Carolina at Chapel Hill, Chapel Hill, NC 27599

**Keywords:** adult neural stem cells, dentate gyrus, cholinergic circuit, diagonal band of Broca, granule cells

## Abstract

Cholinergic neurons in the basal forebrain play a crucial role in regulating adult hippocampal neurogenesis (AHN). However, the circuit and molecular mechanisms underlying cholinergic modulation of AHN, especially the initial stages of this process related to the generation of newborn progeny from quiescent radial neural stem cells (rNSCs), remain unclear. Here, we report that stimulation of the cholinergic circuits projected from the diagonal band of Broca (DB) to the dentate gyrus (DG) neurogenic niche promotes proliferation and morphological development of rNSCs, resulting in increased neural stem/progenitor pool and rNSCs with longer radial processes and larger busy heads. Interestingly, DG granule cells (GCs) are required for DB-DG cholinergic circuit–dependent modulation of proliferation and morphogenesis of rNSCs. Furthermore, single-nucleus RNA sequencing of DG reveals cell type–specific transcriptional changes in response to cholinergic circuit stimulation, with GCs (among all the DG niche cells) exhibiting the most extensive transcriptional changes. Our findings shed light on how the DB-DG cholinergic circuits orchestrate the key niche components to support neurogenic function and morphogenesis of rNSCs at the circuit and molecular levels.

## Significance

Radial neural stem cells (rNSCs) in the adult dentate gyrus (DG) can proliferate and generate new adult-born neurons throughout life contributing to hippocampus-dependent learning and memory. How this process is regulated by distinct neural circuits remains elusive. In this study, we reveal how septo-DG cholinergic circuits orchestrate the key niche cells to support neurogenic function and morphogenesis of rNSCs. Furthermore, using single-nucleus RNA sequencing, we identify cell-specific transcriptional changes in the adult DG in response to cholinergic circuit activity. Our study bridges a long-standing gap in understanding combined circuit and molecular mechanisms underlying activity-dependent regulation of rNSCs.

The cholinergic system in the basal forebrain plays a crucial role in modulating hippocampus-dependent learning and memory (1–4). Within the hippocampus, the dentate gyrus (DG) generates new neurons throughout life via adult hippocampal neurogenesis (AHN). At the critical 4 to 6 wk of cellular age, adult-born neurons possess unique physiological properties that contribute to learning and memory (5–7). Therefore, understanding the role of cholinergic signaling in regulating this process may inform new targets for cognitive improvement.

Previous pharmacological manipulation of cholinergic signaling (cholinesterase inhibitors or selective receptor agonists/antagonists) or neurotoxic lesion of cholinergic neurons suggested that the cholinergic system positively or negatively modulates AHN (reviewed in ref. 8). However, due to the broad actions of these agents on multiple brain regions and cell types, precise circuit and signaling mechanisms underlying cholinergic modulation of AHN remain undefined. In addition, thymidine analogs (such as BrdU or EdU) biased findings toward highly proliferative intermediate progenitor cells, therefore, whether quiescent neural stem cells are subject to cholinergic regulation remains unknown. It has been well established that quiescent radial-glia-like neural stem cells (rNSCs) in adult DG can be induced to self-renew and generate new neurons (9–11). However, it remains unknown whether these key properties of rNSCs are regulated by specific cholinergic circuits. Besides infrequent proliferation, rNSCs also exhibit unique morphology with a long radial process extending and branching into a “bushy head” in the outer granule cell and inner molecular layers (12). Our recent study showed that these bushy processes wrap around glutamatergic synapses formed between mossy cells (MCs) and granule cells (GCs) at these locations, suggesting that busy heads of rNSCs may serve as a functional unit to receive signals from ongoing neural circuits to regulate rNSC function (13). Whether the rNSC morphology is modulated by specific neural circuits remains unexplored. This current study intends to bridge these gaps by addressing circuit-based cholinergic regulation of rNSCs in the adult DG neurogenic niche.

The main source of acetylcholine (ACh) in the hippocampus originates from the septal nuclei, including medial septum (MS) and diagonal band of Broca (DB) (14, 15). Most studies have focused on MS cholinergic neurons on hippocampal CA1 function (16–18); therefore, it remains unknown whether and how septal cholinergic neurons regulate AHN. To address this, we performed anterograde tracing of MS and DB cholinergic neurons and found significantly more DB cholinergic projections to the DG as compared to MS. Importantly, stimulation of the DB-DG (but not MS-DG) cholinergic projections promoted proliferation of rNSCs and increased neural stem/progenitor pool. Furthermore, DB-DG stimulation activated DG mature GCs, which is required for cholinergic regulation of rNSC proliferation and morphology. Our previous studies found that both local and distal neurons can exert direct effects onto rNSCs (10, 19) or indirect effects through interneurons (13, 20) or astrocytes (21). Here, we identify a new circuit mode for rNSC regulation that requires niche GCs to convey cholinergic signals from the distal DB to modulate rNSC function.

To address molecular mechanisms underlying cholinergic circuit modulation of rNSCs, we performed single-nucleus RNA sequencing and identified cell type–specific DG transcriptional changes in response to DB-DG cholinergic stimulation. Together, these findings provide mechanistic insights on how cholinergic circuits orchestrate the DG niche cells to support neurogenic function of rNSCs.

## Results

### Diagonal Band of Broca and Medial Septum Cholinergic Neurons Exhibit Distinct Projection Patterns to the Dentate Gyrus.

**Fig. 1.**
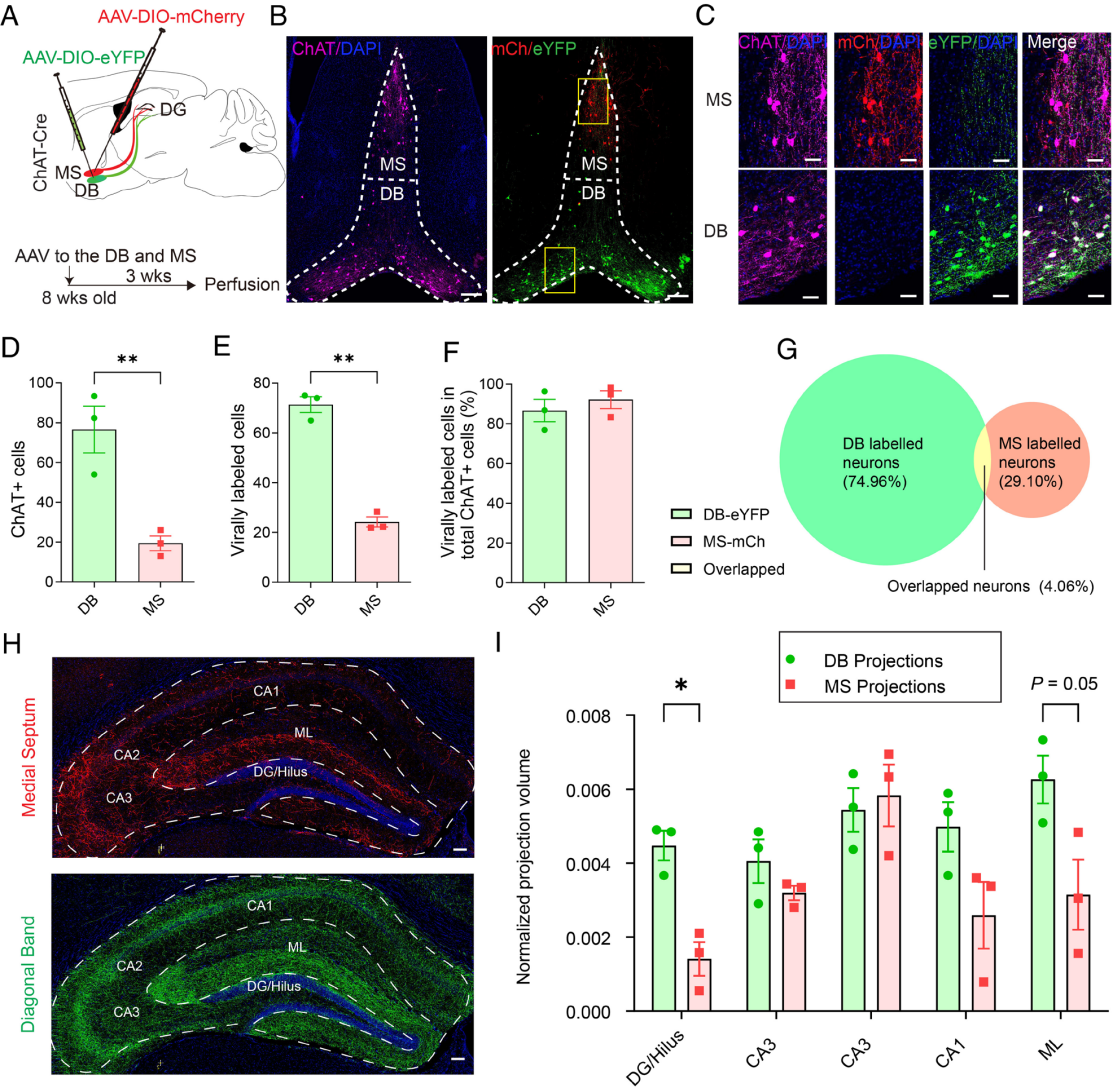
DB and MS cholinergic neurons exhibit distinct projection patterns to the DG. (*A*) AAVs targeting scheme using dual fluorophores (eYFP:DB, mCherry:MS). (*B*) AAV injection to the MS/DB. (Scale bar, 200 µm.) (*C*) Colocalization of ChAT+ cells with reporter fluorophores in the MS and DB. (Scale bar, 50 µm.) (*D*) ChAT+ cells in DB and MS. Each dot represents averaged number of ChAT+ cells from three sections per animal (n = 3 animals). *P* = 0.0097 by Student’s *t* test. (*E*) AAV-labeled cells in DB and MS. Each dot represents averaged number of ChAT+ cells from three sections per animal (n = 3 animals). *P* = 0.0002 by Student’s *t* test. (*F*) Percent AAV-labeled cells per ChAT+ cells in DB and MS (n = 3 animals). (*G*) Venn diagram of AAV-labeled DB cells (Green) and MS cells (Red). Yellow represents overlapped expression. Brackets represent the proportion of each component to the total. (*H*) Hippocampal ROIs drawn around respective subregions, for analysis of (*I*) (Scale bar, 100 µm.) (*I*) Projection densities (normalized to the region volume). MS-DG or DB-DG cholinergic projections were normalized to the number of MS mCherry+ or DB eYFP+ cholinergic neurons. *P* = 0.0072, *q* = 0.0366, by multiple *t* tests with Bonferroni correction. Data were presented as mean ± SEM.

In the basal forebrain, cholinergic neurons are mainly located in the MS and the DB, which send projections to the hippocampal formation (22). We performed anterograde tracing of MS and DB cholinergic neurons with Cre-dependent AAVs expressing mCherry to the MS and eYFP to the DB of ChAT (choline acetyltransferase) - Cre mice (23) (Fig. 1*A* and *SI Appendix*, Fig. S1). Reporter expression was restricted to cholinergic cells expressing ChAT in the MS and the DB (Fig. 1 *B* and *C*). Significantly more eYFP+ ChAT+ neurons were found in the DB than mCherry+ ChAT+ neurons in the MS (Fig. 1 *D* and *E*) with similar labeling efficiency of >80% mCherry+/eYFP+ cells being ChAT+ (Fig. 1*F*), suggesting that DB contains more cholinergic neurons than MS. Of note, MS-DG or DB-DG cholinergic projections were normalized to the number of MS mCherry+ or DB eYFP+ cholinergic neurons. Significantly more DB than MS projections were found in the DG/hilus, along with a trending increase in the molecular layer, without significant changes in CA regions (Fig. 1 *H* and *I*). These results suggest that DB and MS cholinergic neurons exhibit distinct projection patterns to DG and that DB more selectively targets the neurogenic niche. Distinct projection patterns were not due to reporter bias, as switching the colors resulted in similar projection patterns (*SI Appendix*, Fig. S1 *D* and *E*). Due to the close proximity of MS and DB, a small fraction of cells expressed both eYFP and mCherry in MS and DB (~4% of total virally labeled cells) (Fig. 1*G*) and was not expected to exert a significant functional impact on rNSCs.

### The DB-DG Cholinergic Circuits Bidirectionally Regulate rNSC Proliferation.

**Fig. 2.**
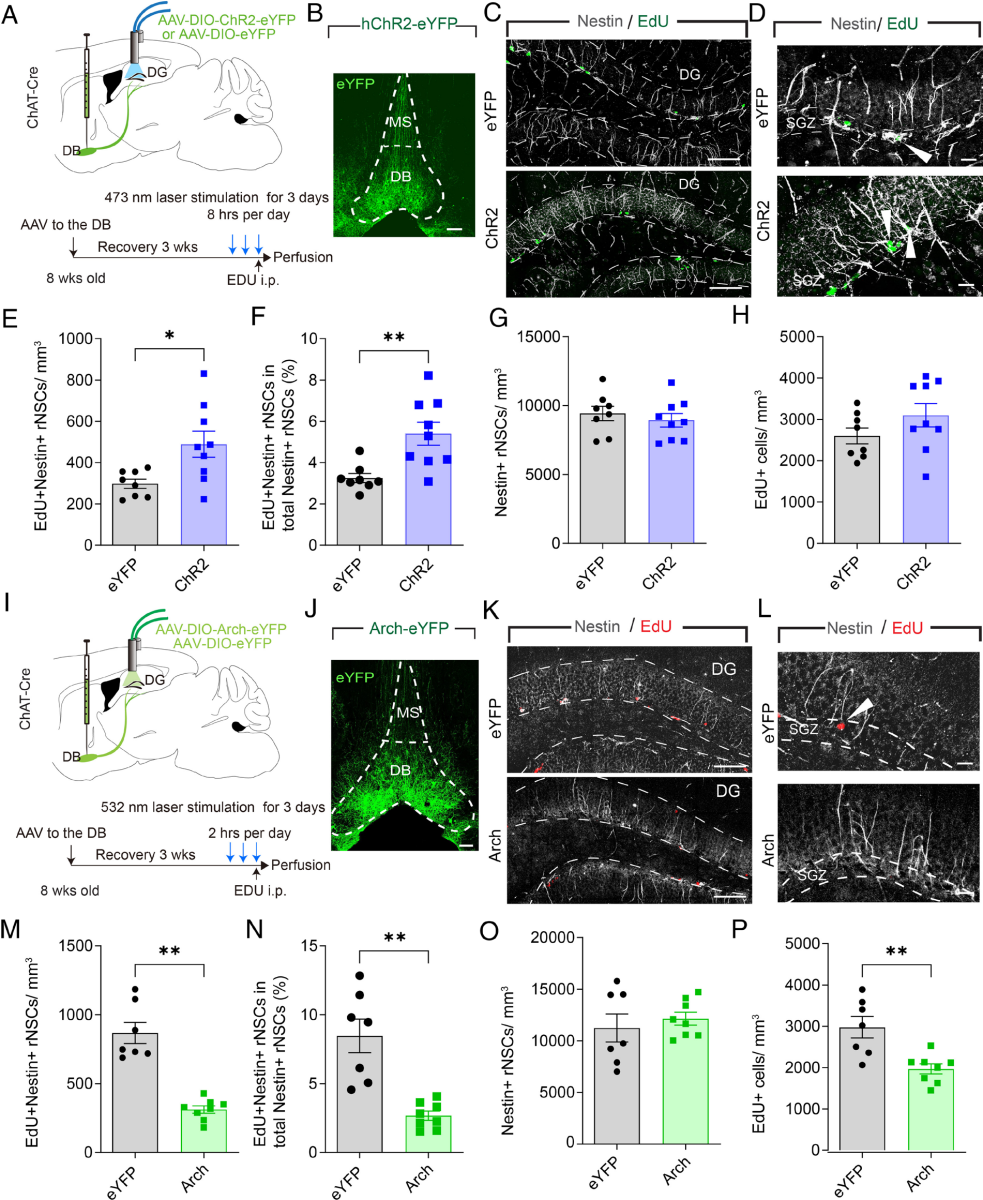
DB-DG cholinergic circuits bidirectionally regulate rNSC proliferation. (*A*) Experimental paradigm. (*B*) DB targeting by AAVs expressing ChR2. (Scale bar, 100 µm.); *Bottom*. (*C*) rNSC proliferation with Nestin and EdU. (Scale bar, 100 µm.) (*D*) Zoomed images of proliferating rNSCs with colocalization of Nestin and EdU (arrowheads.) (Scale bar, 20 µm.) (*E*) Density of proliferating rNSCs. n = 8 animals for eYFP and 9 animals for ChR2. *P* = 0.0155, Student’s *t* test. (*F*) Percent of proliferating rNSCs. n = 8 animals for eYFP and 9 animals for ChR2. *P* =0.0037, Student’s *t* test. (*G*) Density of rNSCs. n = 8 animals for eYFP and 9 animals for ChR2. *P* = 0.05012, Student’s *t* test. (*H*) Density of overall proliferating progeny. n = 8 animals for eYFP and 9 animals for ChR2. *P* = 0.1765, Student’s *t* test. (*I*) Experimental paradigm. (*J*) DB targeting by AAVs expressing Arch3.0. (Scale bar, top,100 µm.) (*K*) rNSC proliferation with Nestin and EdU. (Scale bar, 100 µm.) (*L*) Zoomed images of proliferating rNSCs with colocalization of Nestin and EdU (arrowheads). (Scale bar, 20 µm.) (*M*) Density of proliferating rNSCs. n = 7 animals for eYFP and 8 animals for Arch. *P* = 7.1 × 10^−6^, Student’s *t* test. (*N*) Percent of proliferating rNSCs. n = 7 animals for eYFP and 8 animals for Arch. *P* = 0.0003, Student’s *t* test. (*O*) Density of rNSCs. n = 7 animals for eYFP and 8 animals for Arch. *P* = 0.5364, Student’s *t* test. (*P*) Density of overall proliferating progeny. n = 7 animals for eYFP and 8 animals for Arch. *P* = 0.0028, Student’s *t* test. Data were presented as mean ± SEM.

To determine the functional effects of DB and MS cholinergic inputs on rNSCs, we optogenetically activated DB-DG or MS-DG projections in vivo (8 Hz, 5 ms, 30 s/5 min, 8 h/d for 3 d). Cre-dependent AAVs expressing ChR2 or eYFP were delivered to the DB (Fig. 2 *A* and *B*) or MS (*SI Appendix*, Fig. S2 *A* and *B*) of ChAT-Cre mice. The use of 8 Hz was based on the known firing frequency of forebrain cholinergic neurons during exploration, which relates to the hippocampal theta rhythm mediated by the MS/DB (24–26). Proliferation of rNSCs was assessed by labeling proliferating cells with thymidine analog EdU on the last day of light stimulation. Stimulation of DB-DG cholinergic circuits led to a significant increase in the number of proliferating rNSCs (Nestin+ EdU+) (Fig. 2 *C*–*E*) and the proliferating rate of rNSCs (Fig. 2*F*) without significant changes in the Nestin+ rNSC pool (Fig. 2*G*) and the proliferating progeny (EdU+) (Fig. 2*H*). By contrast, optogenetic activation of MS-DG cholinergic projections did not alter rNSC proliferation (*SI Appendix*, Fig. S2 *C–H*). Similarly, chemogenetic activation of DB cholinergic neurons also led to a significant increase in rNSC proliferation (*SI Appendix*, Fig. S3). These results suggest that activation of DB-DG (but not MS-DG) cholinergic circuits is sufficient for rNSC proliferation.

To address whether the DB-DG cholinergic pathway is required for rNSC proliferation, we optogenetically inhibited DB-DG projections in vivo by delivering Cre-dependent AAVs expressing Arch 3.0 or eYFP in the DB of ChAT-Cre mice (light on 2 h/d for 3 d) (Fig. 2 *I* and *J*). Following EdU injection at the last day of the paradigm, proliferation of rNSCs was assessed. As a result, inhibiting DB-DG cholinergic circuit activity significant decreased the number of proliferating rNSCs (Nestin+ EdU+) (Fig. 2 *K–M*) and the proliferating rate of rNSCs (Fig. 2*N*) without changing the Nestin+ rNSC pool (Fig. 2*O*), along with decreased proliferating progeny (EdU+) (Fig. 2*P*). These results suggest that DB-DG cholinergic circuit activity is required for rNSC proliferation.

### Stimulation of DB-DG Cholinergic Circuits During Early Neurogenesis Stages Leads to Increased Neural Stem/Progenitor Pool.

**Fig. 3.**
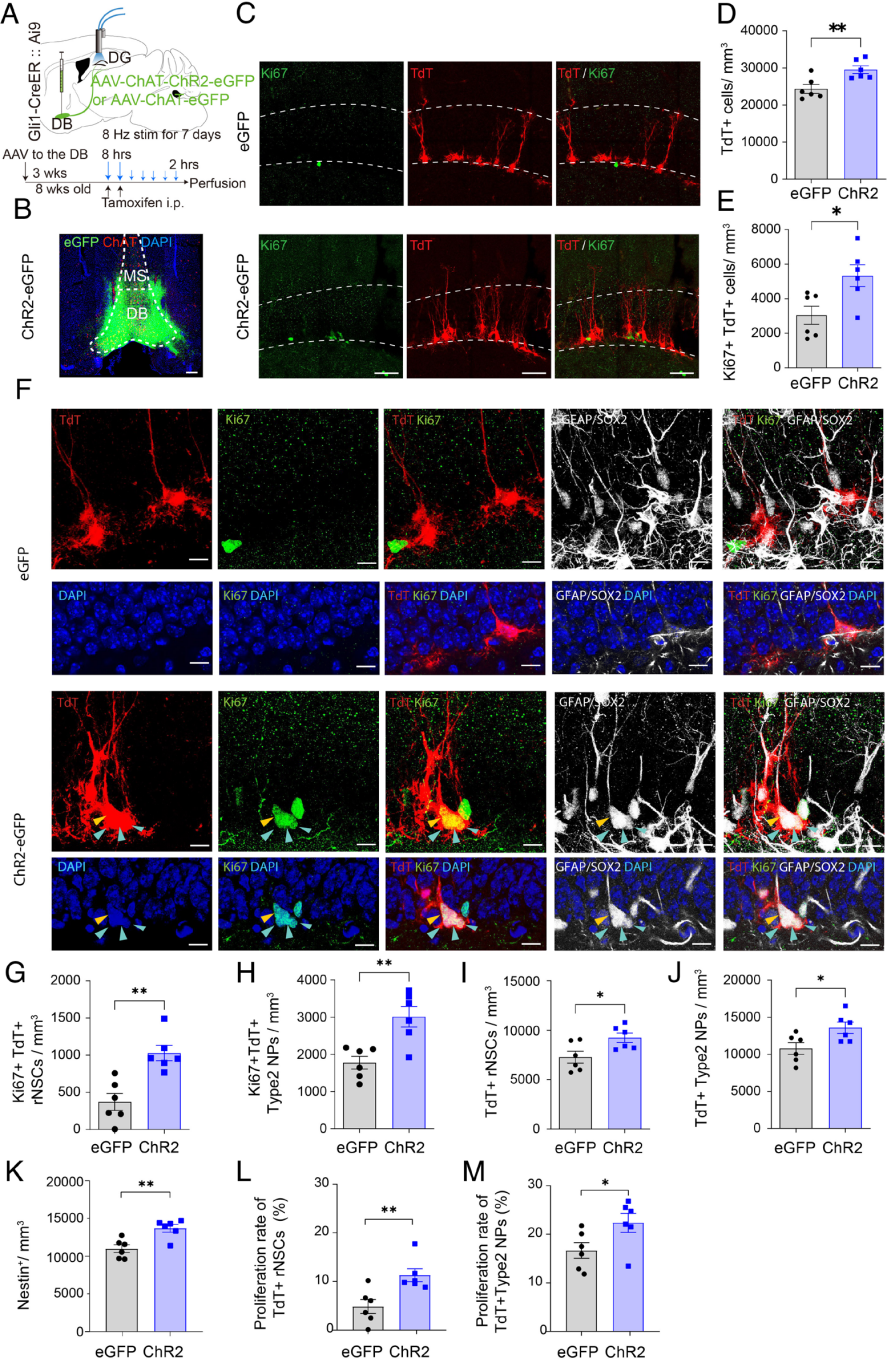
Stimulation of DB-DG cholinergic circuits during early neurogenesis stages leads to increased neural stem/progenitor cells. (*A*) Experimental paradigm. (*B*) AAV-ChAT-ChR2-eGFP expression in DB neurons. (Scale bar, 200 μm.) (*C*) tdTomato+ cells (Red), Ki67+ cells (Green) after optogenetic stimulation of DB-DG cholinergic projections. (Scale bar, 100 μm.) (*D*) Density of tdTomato+ cells. *P* = 0.0078, Student’s *t* test. (*E*) Density of tdTomato+/Ki67+ cells. *P* = 0.019, Student’s *t* test. (*F*) Proliferative/nonproliferative rNSCs and nonradial type 2 NPs in eGFP and ChR2-eGFP groups. rNSCs were identified as tdTomato+ cells containing a GFAP+ radial process. Activated rNSCs coexpress Ki67 (Yellow arrowheads). Type 2 NPs were counted as tdTomato+Sox2+ cells without a GFAP+ radial process. Activated NPs were determined by coexpression of Ki67 (blue arrowheads). (Scale bar, 20 μm.) (*G*) Density of activated rNSCs. n = 6 animals for eGFP and 6 animals for ChR2. *P* = 0.0016, Student’s *t* test. (*H*) Density of proliferating type 2 NPs. n = 6 animals for eGFP and 6 animals for ChR2. *P* = 0.0035 by Student’s *t* test. (*I*) Density of total rNSCs. n = 6 animals for eGFP and 6 animals for ChR2. *P* = 0.0285, Student’s *t* test. (*J*) Density of total type 2 NPs. n = 6 animals for eGFP and 6 animals for ChR2. *P* = 0.0297, Student’s *t* test. (*K*) Density of total rNSCs. n = 6 animals for eGFP and 6 animals for ChR2. *P* = 0.0036, Student’s *t* test. (*L*) Proliferating rate of rNSCs, quantified as Ki67+Tdt+ rNSCs/total Tdt+ rNSCs. n = 6 animals for eGFP and 6 animals for ChR2. *P* = 0.009, Student’s *t* test. (*M*) Proliferating rate of type 2 NPs, quantified as Ki67+Tdt+ Sox2+ NPs/total Tdt+ Sox2+ NPs. n = 6 animals for eGFP and 6 animals for ChR2. *P* = 0.048, Student’s *t* test. Data were presented as mean ± SEM.

To address whether DB-DG cholinergic circuit stimulation regulates the fates of rNSCs and subsequent production of neural stem/progenitor cells, we combined circuit stimulation and indelible tracing of rNSCs using a longer circuit manipulation paradigm. Specifically, we delivered AAV-ChAT-ChR2-eGFP or AAV- ChAT-eGFP (27) to the DB of double transgenic Gli1-CreER::Ai9 mice (Fig. 3 *A* and *B*) to selectively target DG adult rNSCs and early neural progenitors (NPs) with Tdtomato (Tdt) reporter upon tamoxifen (TMX) induced recombination. Three weeks after viral injection, TMX was injected (once daily for 2 d) to induce recombination along with optostimulation of the DB-DG cholinergic pathway for 7 d (8 Hz, 5 ms, 30 s/5 min, 8 h/d for first 2 d, followed by 2 h/d for 5 d). Numbers of Tdt+ and proliferating Ki67+ Tdt+ cells (Fig. 3 *C–E*) were significantly increased. In addition, proliferating Ki67+Tdt+ rNSCs (radial morphology) and proliferating Ki67+Tdt+Sox2+ type 2 NPs (nonradial, horizontal morphology) (Fig. 3 *F–H*) were significantly increased, along with increased pool of Tdt+ rNSCs and type 2 NPs (Fig. 3 *I* and *J*). To ensure that increased rNSC pool was not due to variations in recombination, we also quantified Tdt-negative rNSCs using nestin as an independent marker. An increased number of Nestin+ rNSCs after 7-d circuit stimulation (Fig. 3*K*) confirmed that cholinergic circuit activity increases the pool of rNSCs.

To address whether increased NP pool is a result of increased proliferation of rNSCs, we quantified proliferation rates of rNSCs (proliferating Ki67+Tdt+ rNSCs/total Tdt+ rNSCs) and type 2 NPs (proliferating Ki67+Tdt+Sox2+ NPs/total Tdt+Sox2+ NPs). A significant increase in proliferation rate was observed in rNSCs (with only marginal increase for type 2 NPs) (Fig. 3 *L* and *M*). This suggests that cholinergic circuit induced neurogenic effects likely result from increased neurogenic proliferation of rNSCs.

### Dentate Granule Cells are Required for Cholinergic Circuit Activity-Dependent Regulation of rNSC Proliferation.

**Fig. 4.**
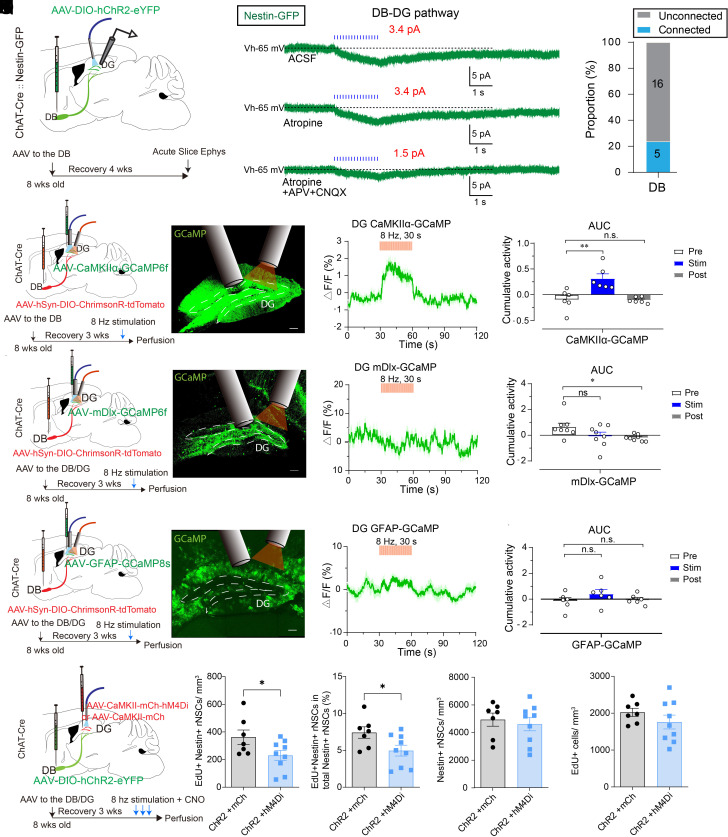
DG GCs are required for cholinergic circuit activity-dependent regulation of rNSC proliferation. (*A*) Electrophysiological recording scheme from a Nestin-GFP-positive cells. (*B*) Light evoked inward current in GFP+ rNSCs upon optogenetic stimulation (10 Hz, 2 s) of DB-DG cholinergic projections. (*C*) Light-evoked inward current in GFP^+^ rNSCs in the presence of atropine (10 µM). (*D*) Light-evoked inward current in GFP^+^ rNSCs in the presence of atropine (10 µM), APV (100 µM), and NBQX (20 µM). (*E*) Proportion of Nestin-GFP cells functionally connected to DB Cholinergic afferents. (*F, G*, and *H*) Experimental paradigm for optogenetic stimulation of DB-DG cholinergic projections and simultaneous photometry recording of GCs (*F*), interneurons (*G*), or astrocytes (*H*). (*I, J*, and *K*) Expression of AAV-CaMKII-GCaMP6f (*I*), AAV-mDlx-GCaMP6f (*J*), AAV-GFAP-GCaMP8s (*K*) along with optic fiber implantation in the DG. (Scale bar, 100 μm.) (*L, M*, and *N*) Population calcium activity of DG GCs (*L*), interneurons (*M*), or astrocytes (*N*) in response to optogenetic stimulation of DB-DG cholinergic projections. (*L*) n = 31 episodes from 6 animals; (*M*) n = 43 episodes from 8 animals; (*N*) n = 22 episodes from 6 animals. (*O, P*, and *Q*) Average fluorescence of DG cells before, during, and after optostimulation of DB-DG cholinergic projections. (*O*) GCs, n = 6 animals, one-way ANOVA between each state, *F*_2,15_ = 10.81, *P* < 0.001, followed by Dunnett’s multiple comparisons test; (*P*) interneurons, n = 8 animals, *F*_2,21_ = 3.37, *P* = 0.0537; (*Q*) astrocytes, n = 6 animals, *F*_2,15_ = 1.18, *P* =0.3346. (*R*) Experimental paradigm. (*S*) Density of proliferating rNSCs. n = 7 animals for mCherry and 9 animals for hM4Di. *P* = 0.0489 by Student’s *t* test. (*T*) Percent of proliferating rNSCs. n = 7 animals for mCherry and 9 animals for hM4Di. *P* = 0.0386 by Student’s *t* test. (*U*) Density of rNSCs. n = 7 animals for mCherry and 9 animals for hM4Di. (*V*) Density of overall proliferating progeny. n = 7 animals for mCherry and 9 animals for hM4Di. Data were presented as mean ± SEM.

We previously established a close correlation between rNSC activity and proliferation state upon selective manipulation of distinct neural circuits. Specifically, increased rNSC proliferation correlates with depolarization of rNSCs (13, 19, 21). Therefore, we sought to address whether DB-DG cholinergic circuits impact the activity of rNSCs, through either direct or indirect mechanisms. To investigate this, we performed patch-clamp recording of rNSCs in acute brain slices prepared from ChAT-Cre::Nestin-GFP mice injected with AAV-DIO-ChR2-mCherry in the DB (Fig. 4*A*). Optogenetic activation of DB-DG projections induced inward currents in a portion of recorded rNSCs (Fig. 4 *B* and *E*). Interestingly, light-evoked currents were not affected by atropine, a muscarinic ACh receptor antagonist (Fig. 4*C*). Instead, they were largely blocked by ionotropic glutamate receptor (iGluR) antagonists AP5 (for NMDARs) and CNQX (for AMPARs) (Fig. 4*D*), suggesting that iGluRs (but not mAChRs) mediate cholinergic activity-induced depolarization of rNSCs. These results indicate that DB-DG cholinergic circuits require a glutamatergic intermediary to relay signals to rNSCs.

We next sought to determine the niche cells recruited by DB-DG cholinergic circuits. Septo-hippocampal cholinergic circuits exert broad innervations onto various DG cell types, including GCs, interneurons, and astrocytes (28–30). Therefore, we recorded the population calcium activity of GCs, interneurons, and astrocytes by fiber photometry upon optostimulation of the DB-DG cholinergic pathway. Cre-dependent AAVs expressing red-shifted ChR2 (ChrimsonR) was injected to the DB, along with injection of various forms of AAVs to the DG of ChAT-Cre mice: AAV-CaMKII-GCaMP6f (for labeling GCs), AAV-mDlx-GCaMP6f (for labeling interneurons), or AAV-GFAP-GCaMP8s (for labeling astrocytes). Interestingly, GCs (but not interneurons and astrocytes) significantly increased their calcium activity upon optogenetic stimulation of the DB-DG cholinergic projections (Fig. 4 *F*–*Q*). Similarly, cFos expression in GCs (*SI Appendix*, Fig. S4 *A* and *B*) was significantly increased. DG interneurons exhibited a small but significant decrease in population calcium activity following cholinergic circuit stimulation. Septo-hippocampal cholinergic projections have been shown to lower the threshold for GC firing via interneuron-mediated disinhibition (31). Therefore, increased GC activity induced by cholinergic circuit activation may reflect the net effects from both direct cholinergic modulation of GCs and indirect modulation mediated by interneurons.

To directly address whether GCs are required for cholinergic modulation of rNSCs in vivo, we chemogenetically inhibited dentate mature GCs labeled with AAV-CaMKII-hM4Di/mCherry upon optogenetic activation of DB-DG cholinergic projections by delivering Cre-dependent AAVs expressing ChR2 to the DB of ChAT-Cre mice (Fig. 4*R*). This manipulation led to a significant decrease in the number of proliferating rNSCs and the proliferation rate of rNSCs in ChAT-Cre mice injected with both ChR2 and hM4Di as compared to ChR2 and mCherry controls (Fig. 4 *S* and *T*) without significantly altering the number of EdU+ proliferating progeny or Nestin+ rNSCs (Fig. 4 *U* and *V*). While AAVs with the CaMKII promoter label both GCs and MCs, our previous study showed that selective inhibition of MCs increases rNSC proliferation, presumably through disinhibition of GCs resulted from inhibition of MC-interneuron pathway (13). Therefore, decreased rNSC proliferation upon inhibition of both GCs and MCs upon cholinergic circuit stimulation was most likely mediated by inhibition of GCs. This suggests that GCs are required for cholinergic regulation of rNSCs.

### Dentate Granule Cells are Required for Cholinergic Circuit Activity-Dependent Regulation of rNSC Morphogenesis.

**Fig. 5.**
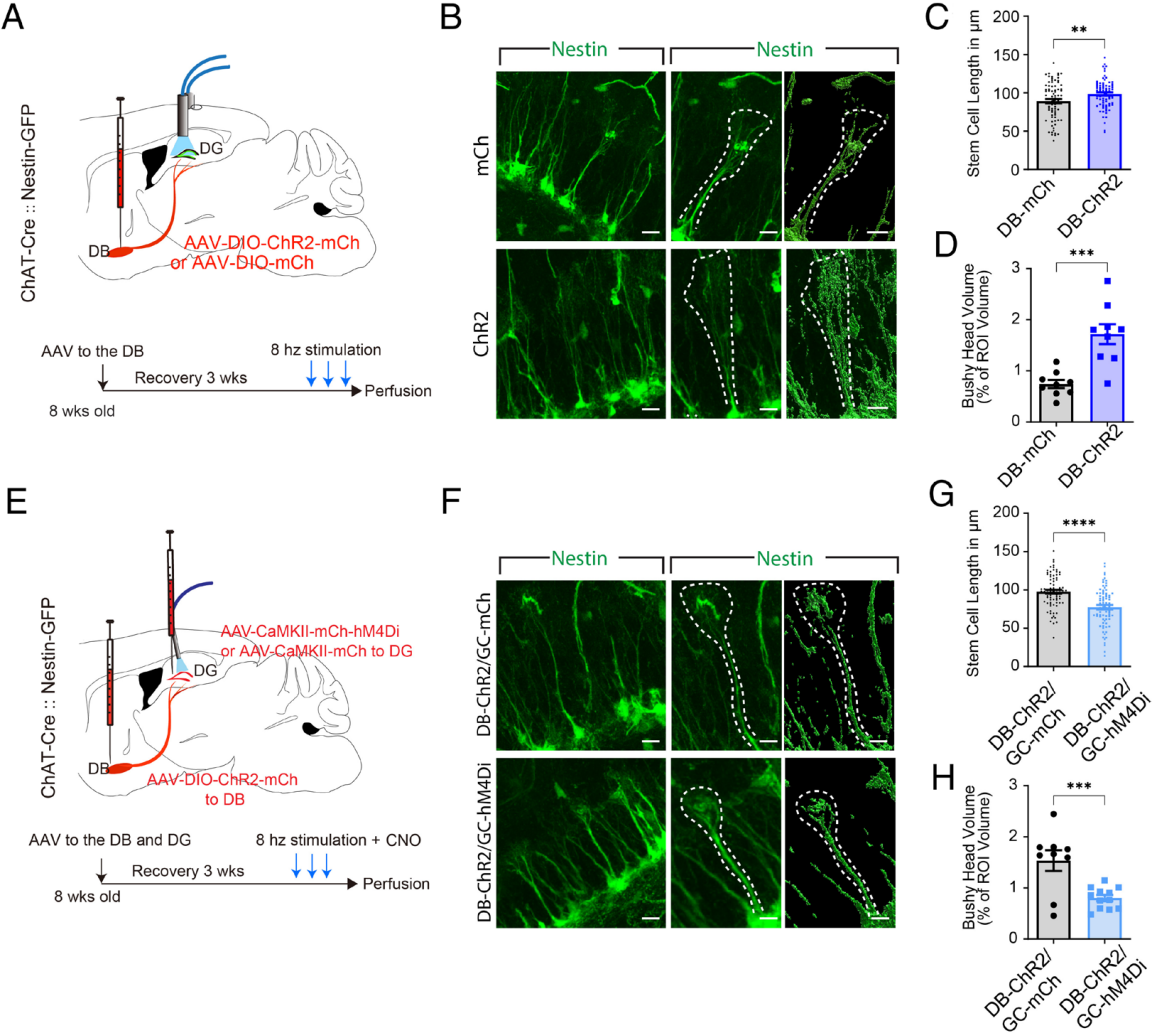
Dentate GCs are required for cholinergic circuit activity-dependent regulation of rNSC morphogenesis. (*A*) Experimental paradigm. (*B*) rNSC bushy heads from a ChAT-Cre::Nestin-GFP animal after optogenetic activation of DB-DG cholinergic terminals. (*Left* Scale bar, 20 µm, *Right* Scale bar, 2 µm.) (*C*) Mean length of rNSCs. mCherry: n = 80 cells from 3 animals; ChR2-mCherry, n = 80 cells from 3 animals, *P* = 0.0074. (*D*) Volume of rNSC bushy heads. mCherry: n = 9 slices from 3 animals; ChR2-mCherry, 9 slices from 3 animals, *P* = 0.0003. (*E*) Experimental paradigm. (*F*) rNSC bushy heads from a ChAT-Cre::Nestin-GFP animal after simultaneous chemogenetic inhibition of DG GCs and optogenetic activation of DB-DG cholinergic terminals. (*Left* Scale bar, 20 µm, *Right* Scale bar, 2 µm.) (*G*) Mean length of rNSCs. mCherry: n = 80 cells from 3 animals; ChR2-mCherry, n = 80 cells from 4 animals, *P* < 0.0001. (*H*) Volume of rNSC bushy heads. mCherry: n = 9 slices from 3 animals; hM4Di: n = 12 slices from 4 animals, *P* = 0.0010. Data were presented as mean ± SEM.

The rNSCs are heterogeneous (12) and extend a radial process that terminates as a bushy head in the outer granule cell and inner molecular layers. We examined the length of the radial processes in the predominant population of rNSCs (Type α cells, ~75% of the total rNSC population) with long radial processes extending and branching in the inner molecular layer (IML). Using ChAT-Cre::Nestin:GFP mice injected with AAVs expressing Cre-dependent ChR2 or mCherry in the DB (Fig. 5*A*), we found that optogenetic activation of DB-DG cholinergic projections led to both increased length of the radial processes and increased size of bushy heads in rNSCs (Fig. 5 *B*–*D*).These morphological alterations were replicated in Gli1-CreER::Ai9 mice after the same circuit manipulation (*SI Appendix*, Fig. S4 *C*–*E*).

To address whether GCs are required for cholinergic activity-dependent regulation of rNSC morphology, we inhibited mature GCs during optogenetic stimulation of DB-DG cholinergic circuits. Specifically, Cre-dependent AAVs expressing ChR2 was delivered to the DB and AAVs expressing CaMKII-hM4Di/mCherry were delivered to the DG of ChAT-Cre::Nestin-GFP mice for simultaneous optostimulation of DB-DG projections and chemogenetic inhibition of GCs (Fig. 5*E*). We found significantly decreased length of radial processes and size of bushy heads in rNSCs in ChAT-Cre::Nestin-GFP mice injected with both ChR2 and hM4Di as compared to controls injected with ChR2 and mCherry (Fig. 5 *F*–*H*).This suggests that GCs are required for cholinergic circuit activity-dependent regulation of rNSC morphogenesis.

### Dentate Granule Cells Exhibit the Most Extensive Transcriptional Changes in Response to Cholinergic Circuit Stimulation.

**Fig. 6.**
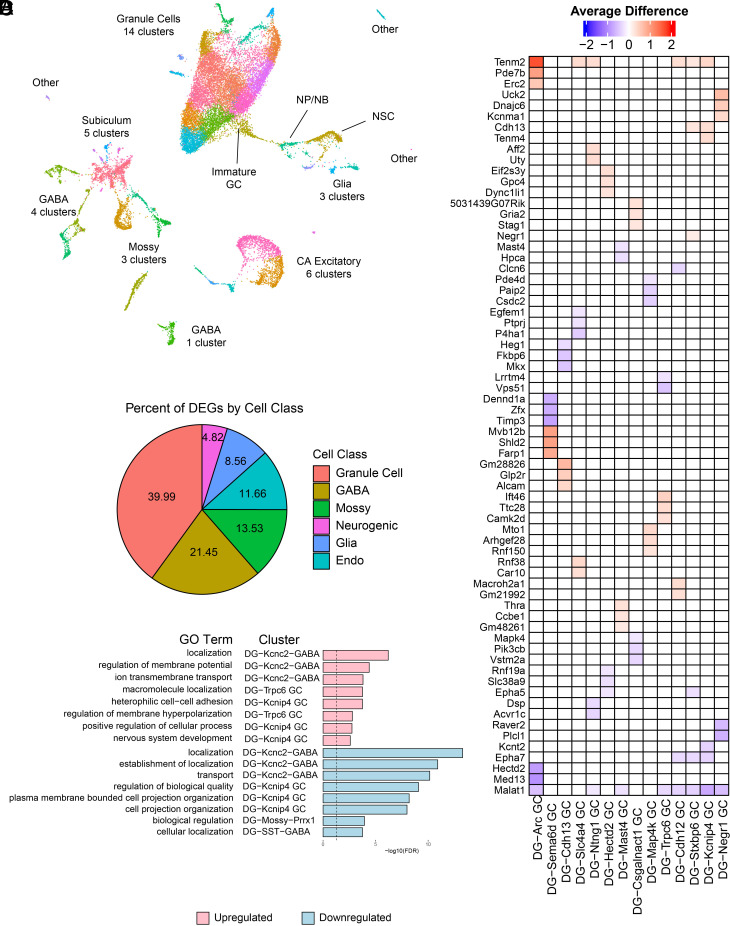
DG GCs exhibit the most transcriptional changes in response to cholinergic circuit stimulation. (*A*) UMAP dimension reduction analysis of 42 DG clusters identified from snRNA-seq. (*B*) Percentage of DEGs by cell class. (*C*) Heatmap of the most significant DEGs in each direction (Wilcoxon rank-sum test *P* < 0.01, log_10_-fold change > 0.1). (*D*) Most significant GO terms from the top two most responsive neuronal clusters. Up-regulated (pink) and down-regulated (light blue) with −log_10_(FDR) > 1.5.

To mechanistically explore how cholinergic circuits recruit GGs to regulate rNSCs, we used single-nucleus Split Pool Ligation-based Transcriptome sequencing (SPLiT-seq) as an unbiased approach in a cell type–specific manner (*SI Appendix*, Fig. S5). Uniform Manifold Approximation and Projection (UMAP) analysis uncovered all known major DG cell types (32–36), including mature GCs, interneurons (GABA), mossy cells (MCs), astrocytes, oligodendrocytes (oligo), oligodendrocyte progenitor cells (OPCs), microglia, endothelial cells, along with the neurogenic cell clusters related to AHN, including NSCs, neural progenitors/neuroblasts (NPs/NBs), and immature GCs (33, 35, 37) (Fig. 6*A* and *SI Appendix*, Figs. S6 and S7). Differential gene expression analysis revealed a total of 2,676 differentially expressed genes (DEGs) between the stimulated and unstimulated animals (Fig. 6*B* and Dataset S1). The majority were found in mature neuron clusters (2,006 DEGs, 75%). By contrast, non-neuronal cells exhibited much fewer DEGs in response to cholinergic circuit activation (670 DEGs, 25%) (Fig. 6*B*). Among mature neurons, GCs exhibit most DEGs (1,070), followed by interneurons (574). These data support the fiber photometry results in which GCs were the most responsive population to optogenetic stimulation of the DB-DG cholinergic pathway, followed by interneurons (Fig. 4).

In addition, single-nucleus RNA sequencing (snRNA-Seq) data revealed cell populations expressing cholinergic receptors and could directly respond to DB stimulation. Chrm1 and Chrnα7 were expressed in all GC clusters (*SI Appendix*, Fig. S8). Interestingly, PV interneurons exhibited high-level expression of Chrm2 (Gi-coupled) (*SI Appendix*, Fig. S8), while Cnr1 interneurons express high-level Chrm3 (Gq-coupled), suggesting that cholinergic circuit stimulation may simultaneously excite or inhibit distinct subpopulations of interneurons. Curiously, interneuron responses to cholinergic stimulation are heterogeneous, as only 6.8% of DEGs overlap across distinct interneuron clusters, as compared to GC clusters with 31.4% overlapping DEGs. Further dissection of distinct interneuron subpopulations in response to cholinergic circuit stimulation will provide valuable information on the functional interaction among them.

Among up-regulated DEGs in GCs (Fig. 6*C*), *Tenm2* was identified as one of the top three most up-regulated genes in several GC clusters (6/14). *Tenm2* encodes the teneurin 2 transmembrane glycoprotein that can be released from the cell surface and is involved in synaptogenesis, neurite outgrowth, axon guidance, and neuronal connectivity, through cell–cell contact (38–40). Another is Cdh13, a cell adhesion protein involved in synaptic function (41). Expression of Cdh13 was validated by immunohistology and confirmed increased expression in GCs from stimulated mice as compared to the controls (*SI Appendix*, Fig. S9). Gene Ontology (GO) terms with up-regulated DEGs in GCs and interneurons are related to localization (protein localization to the cell surface and axons), regulation of membrane potential, ion transmembrane transport, and cell–cell adhesion via membrane adhesion molecules. GO terms with down-regulated DEGs in GCs and interneurons are related to localization, transport, regulation of biological quality, cell projection organization, synapse organization, and regulation of protein metabolic process (Fig. 6*D* and Dataset S2). These findings suggest that cholinergic circuits may modulate activity and synaptic function of GCs and interneurons through diverse mechanisms of ion channels, transporters, receptors, and cell adhesion molecules.

### Cholinergic Circuit Stimulation Induces Molecular Changes in rNSCs Related to Activity, Proliferation, and Morphogenesis.

**Fig. 7.**
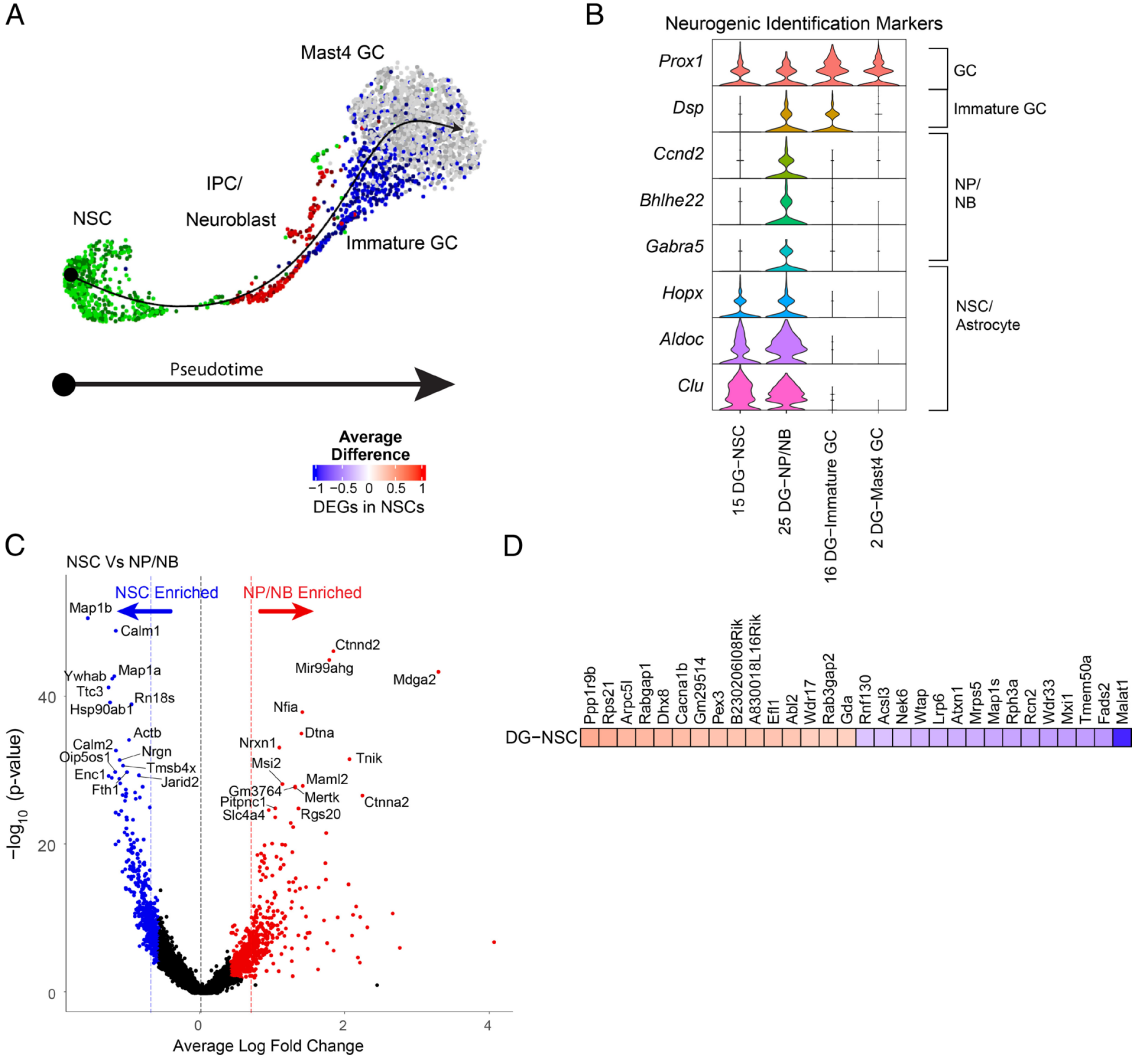
Cholinergic circuit stimulation induces transcriptomic changes in rNSCs related to activity, proliferation, and morphogenesis. (*A*) Slingshot pseudotime analysis of clusters from the neurogenic cell class and the most proximal GC cluster. (*B*) Marker gene expression in neurogenic cell class clusters, from refs. 33, 35, and 37. (*C*) Differentially up-regulated genes in the combined, both eYFP (control) and ChR2 (treatment) rNSC cluster (red) and NP/NB cluster (blue). (*D*) Ten most significant up-regulated and down-regulated DEGs in the rNSC cluster.

Finally, we examined cholinergic circuit activity-induced DEGs in rNSCs. Pseudotime analysis using Slingshot (42) (*SI Appendix*, Fig. S10) revealed transitions from the rNSC cluster to the NP/NB cluster and the immature GC cluster, and finally to the *Mast4*+ mature GC cluster (Fig. 7 *A* and *B*), in agreement with known neurogenic lineage progression of these cells. Comparatively, *Mertk*, a TAM receptor that supports neural stem cell survival and proliferation (43), was identified in rNSCs; and *Calm1* and *Calm2*, two calmodulin proteins that aid neuroblast migration, were found in the NP/NB cluster (44) (Fig. 7*C*). Together, these results confirmed the classification of our neurogenic clusters along a developmental lineage.

In the rNSC cluster, 61 DEGs were identified, (36 up and 25 down) (Fig. 7*D*), including DEGs associated with activity (Cacna1b), generation of new neurons (Atxn1, Mapk8, Atp7a, Acsl3, and Tenm2), as well as actin-mediated structural development (Abi1, Abl2, Mapk8, and Atp7a). Together, these transcriptional signatures provide the molecular basis for our findings that optogenetic activation of the DB-DG cholinergic pathway led to increased rNSC proliferation (Fig. 2) and activity (Fig. 4), as well as altered morphogenesis in rNSC structure (Fig. 5).

## Discussion

Our study bridged several knowledge gaps in the current understanding of cholinergic modulation of AHN. First, previous studies using pharmacological agents or neurotoxic lesion to alter cholinergic system lack specificity, as ACh exerts broad and complex actions on multiple brain regions and cell types. Here we directly manipulated septal cholinergic projections from the DB to the DG, thus limiting the manipulation within the neurogenic niche. Second, thymidine analogs typically label proliferating neural progenitors, and whether slowly proliferating rNSCs are subject to direct or indirect cholinergic regulation has been unknown. Our findings support a model that DB-DG cholinergic circuits indirectly modulate adult rNSC function through GC intermediaries. Third, several recent studies using slice electrophysiology to dissect the cholinergic influence on DG have generated conflicting results (28–30). One found that GCs were inhibited upon optogenetic stimulation of MS/DB cholinergic projections through cholinergic activation of astrocytes that in turn activate interneurons (30). Two others found that stimulating the septo-hippocampal cholinergic pathway lowered the threshold for GC firing thus making them more excitable, potentially through cholinergic activation of GCs and inhibition of interneurons that in turn contributes to disinhibition of GCs (28, 29). Therefore, circuit-based cholinergic modulation of hippocampal neurogenesis in vivo remains unexplored. Our study used in vivo approaches and showed that activation of septo-DG cholinergic circuits promotes rNSC proliferation through mature GCs. Fourth, both MS and DB send cholinergic projections to DG, however, their projection patterns and relative contributions to AHN remain elusive. Our study demonstrated that activation of DB-DG (but not MS-DG) cholinergic projections promotes quiescent rNSC activation and increases the neural stem/progenitor pool. Fifth, rNSCs exhibit unique morphology with a long radial process branching and forming bushyhead structures at the hot spot where active circuit inputs are abundant. Whether specific neural circuits regulate their morphological development has never been investigated. In this study, we showed that GC activity is required for mediating the cholinergic circuit–dependent increase in the length of radial processes and increased volume of bushy heads in response to the DB-DG cholinergic circuit stimulation. Sixth, septal cholinergic neurons can exert broad and complex cholinergic influence on multiple DG cell types, however, which niche cell types play a key role in mediating cholinergic activity-dependent rNSC regulation remained unknown. Our study revealed GCs as a key intermediate to convey cholinergic signals to rNSCs. This is supported by snRNA-seq of DG showing that GCs (among all the niche cells) exhibit the most extensive transcriptional changes in response to the DB-DG cholinergic circuit stimulation. Although single-cell/nucleus RNA-seq has been extensively performed in various brain regions including the DG, our study made initial attempt to profile transcriptional changes induced by specific neural circuits. Knowledge gained from our studies provides a framework to investigate the functional impact and therapeutic relevance of cell type–specific changes in response to altered cholinergic activity under various physiological and pathological conditions.

One unexpected finding is that cholinergic circuit activity-induced rNSC activation requires GCs. This is consistent with our snRNA-Sequencing data showing the low expression level of ACh receptors in rNSCs. This study adds another example to our growing evidence supporting the critical role of glutamate-releasing niche cells in mediating rNSC activation. Specifically, glutamate-releasing niche cells either directly signal rNSCs, such as mossy cells (13), or serve as a relay to mediate signals from other cells, such as astrocytes (21, 30). Our snRNA-seq data revealed that cholinergic activity-induced DEGs are associated with the morphological development of radial processes/bushy heads of rNSCs. Our recent electron microscopy analyses showed that bushy heads of the rNSCs express NMDA receptors and wrap around glutamatergic synapses (13), suggesting that the bushy heads of rNSCs could serve as a glutamate sensors, which pass the signals down to the somas to induce their neurogenic proliferation. Interestingly, in response to the DB-DG cholinergic circuit stimulation, rNSCs exhibit significant structural remodeling with longer radial processes and larger bushy heads, which could prime them to receive more niche signals critical for their proliferation. Future studies using live imaging to simultaneously track the bushy head dynamics and proliferation status of rNSCs upon cholinergic circuit stimulation will help address this interesting structure–function relationship in rNSCs.

In addition to glutamate, activated GCs have been shown to regulate adult hippocampal rNSCs and neurogenesis by secreting niche factors, such as secreted frizzled-related protein 3 (sFRP3), a Wnt inhibitor (45), or through cell–cell contact signals, such as ephrin-B3–EphB2 signaling (46). To address this question further, we performed NicheNet analysis to investigate the ligand–receptor interaction between GCs and rNSCs (47). Interestingly, among all the GC clusters, cholinergic circuit–activated GC cluster (Arc-GC) appeared to possess several ligands that interact with receptors in rNSCs, including Dscam, Inhba, and Sdk1 (*SI Appendix*, Fig. S11). Whether these secretory or membrane-bound ligands from cholinergic circuit–activated GCs play functional roles in rNSC regulation requires further investigation.

## Materials and Methods

### Animals.

Single or double transgenic adult mice (8 to 10 wk, males and females) were used and randomly assigned to experimental groups. The following mouse lines were obtained from the Jackson Laboratory: ChAT-IRES-Cre, Gli1-CreER, and Ai9. Nestin::GFP mice (48) were obtained from Dr. Grigori Enikolopov at Stony Brook University. All the mice used for this study are heterozygous for transgene expression. All animal procedures followed the NIH Guide for Care and Use of Laboratory Animals and were approved by the Institutional Animal Care and Use Committee at the University of North Carolina at Chapel Hill.

### Stereotaxic Injections.

Stereotaxic injections were performed as described in ref. 49. Detailed information on the coordinates for stereotaxic delivery of AAVs and optic fiber placement is included in *SI Appendix*, *SI Materials and Methods*.

### Fiber Photometry Recording.

Photometry recordings were performed as described previously (50). Briefly, recordings were carried out in an open-top home cage (21.6” 17.8” 12.7 cm) in a 30 lx red light environment. Photometry data were exported to MATLAB R2022b for analysis. Photometry signals (△F/F) were derived by calculating (F–F0)/F0, where F0 is the mean of the fluorescence signal. Cumulative activity = △F/F × Time. More detailed information is included in *SI Appendix*, *SI Materials and Methods*.

### Optogenetic Manipulation.

For activation, animals were stimulated for 3 d (8 Hz, 1 ms, 30 s/5 min, 8 h/d, 473 nm laser at 5 mW). For inhibition, animals were stimulated for 3 d (continuous light on for 2 h/d 532 nm laser at 5mW). For lineage tracing combined with optostimulation, animals were stimulated for 7 d (first 2 d: 8 h/d; the following 5 d: 2 h/d, light paradigm: 8 Hz, 1 ms, 30 s/5 min). For optogenetic stimulation during fiber photometry recording, animals were stimulated for 30 s (8 Hz, 1 ms).

### Chemogenetic Manipulation.

For neurogenesis experiments, we applied CNO to activate DB cholinergic neurons through drinking water (3 d, 2.5 mg/200 ml). For analyses of morphology and proliferation of rNSCs upon optogenetic stimulation of cholinergic circuits, we inhibited DG GCs through intraperitoneal injection of CNO (1 mg/kg). Detailed information is included in *SI Appendix*, *SI Materials and Methods*.

### EdU and Tamoxifen Administration.

To label proliferating cells, we administered thymidine analog EdU through intraperitoneal injection (4 mg/kg). For lineage tracing, we administered TMX through intraperitoneal injection (80 mg/kg) to induce recombination in Gli1-CreER::Ai9 mice. Detailed information is included in *SI Appendix*, *SI Materials and Methods*.

### Immunohistology, Image Acquisition, and Imaging Analysis.

Immunohistology was performed as described in ref. 49. The following primary antibodies were used at the following concentrations: goat anti-GFP 1:1000 (Rockland), rabbit anti-RFP 1:1000 (Rockland), goat anti-ChAT 1:800 (Millipore AB144P), chicken anti-Nestin 1:250 (Aves NES), rabbit anti-c-Fos 1:5000 (Santa Cruz Biotechnology SC-52), rabbit anti-Cdh-13 1:1000 (LSBio LS-B481), rabbit anti-Ki67 1:500 (Invitrogen #PA5-19462), goat anti-Sox2 1:500 (R&D AF2018), and goat anti-GFAP 1:2000 (Sant Cruz Biotechnology Sc-6170). Sox2 and GFAP were both placed in the 647 channel, as Sox2 is exclusively nuclear whereas GFAP is exclusively cytoplasmic (19). Images were obtained using an Olympus FV3000 microscope. Imaging analysis was performed using Fiji/ImageJ and Imaris. Detailed information on the quantifications of anterograde tracing of cholinergic projection, neurogenesis and morphology of rNSCs, and viral labeling of MS/DB is included in *SI Appendix*, *SI Materials and Methods*.

### Slice Electrophysiology.

At 4 to 5 wk after AAV5-EF1a-DIO-hChR2(H134R)-eYFP injections, ChAT-Cre::Nestin-GFP mice were killed for acute slice preparation, as described previously (10, 13, 19–21). We recorded light-evoked currents of nestin-GFP rNSCs under the voltage-clamp recordings (holding at −65 mV) upon optostimulation of DB-DG cholinergic projections (8 Hz light pulses for 2 s at every 30 s). To block NMDA, AMPA/kainate, and muscarinic ACh receptors, respectively, 100 μM d-(−)-2-amino-5-phosphonopentanoic acid (d-APV), 20 μM 6-cyano-7-nitroquinoxaline-2,3-dione (CNQX), and 10 μM atropine were bath applied. Detailed methods on slice preparation and patch-clamp recording are included in *SI Appendix*, *SI Materials and Methods*.

### Single-Nucleus RNA-Seq.

Animals were injected with AAV5-EF1α-DIO-hChR2(H134R)-EYFP or AAV5-EF1α-DIO-EYFP, followed by optogenetic stimulation described above. Dorsal DGs from 10-wk-old animals (5 eYFP control, 5 ChR2) were microdissected and processed for single nucleus SPLiT-seq with minor modifications (51). Nuclei isolated from flash-frozen DGs were performed as previously described (52, 53). FASTQ files were deconvoluted, and transcript abundance was estimated using zUMIs v2.9.6 (54) and GENCODE vM26 annotations (55). After barcoding and sequencing, >20 k cells passed quality control (10,072 control cells; 11,575 ChR2 cells). Following sequencing, individual libraries were integrated using Seurat V3, and unbiased cell clusters were assigned via Louvain–Jaccard clustering with multilevel refinement. Detailed methods for library preparation and sequencing analysis are included in *SI Appendix*, *SI Materials and Methods*.

### Inference of Developmental Trajectories.

Pseudotime trajectory of analysis was performed with Slingshot (42), filtering out lowly expressed genes and using a two-dimensional embedding based on PCA of Z-scores of gene expression patterns in rNSC, rNSC-Like, and Neuroblast cells in control and animals

### Niche Net Analysis.

NicheNet analysis was performed following the established pipeline in ref. 47. Analysis was performed by selecting the rNSC cluster as the receiver, and GCs as the senders. Top ligand and receptor pairs were identified by using existing DEGs in each GC cluster.

### Statistical Methods.

All statistics were performed using R version 4.1, Seurat Version 3.1 or 4, or PRISM 9. Individual animals were treated as biological replicates unless otherwise noted in figure legends. Data were all presented as mean ± SEM by animals unless otherwise noted in figure legends. No data were excluded unless specifically specified.

## Supplementary Material

Appendix 01 (PDF)

Dataset S01 (XLSX)

Dataset S02 (XLSX)

## Data Availability

Single-nucleus RNA-seq data have been deposited to the Gene Expression Omnibus (GEO) under accession GSE202481 (56). Code is available in a Song Lab Repo on GitHub at https://github.com/zekachen/Chen_Quintanilla_cholinergic_neurogenesis2024 (57).
