# Supplementary material for: Septo-dentate gyrus cholinergic circuits modulate function and morphogenesis of adult neural stem cells through granule cell intermediaries
Source: Proc Natl Acad Sci U S A. 2024 Sep 23;121(40):e2405117121. doi: 10.1073/pnas.2405117121 (PMC11459179; doi:10.1073/pnas.2405117121)
Supplement: Supplementary file 1 — Appendix 01 (PDF) [file pnas.2405117121.sapp.pdf]

Supporting Information for
Septo-dentate gyrus cholinergic circuits modulate function and morphogenesis of adult neural stem cells through granule cell intermediaries

Ze-Ka Chen[a, b, 1], Luis Quintanilla[a, b, c, 1], Yijing Su[d, e], Ryan N Sheehy[a, b, f], Jeremy M. Simon[b, g, h], Yan-Jia Luo[a, b], Ya-Dong Li[a, b], Zhe Chen[a, b], Brent Asrican[a, b], Dalton S Tart[a], W. Todd Farmer[a], Guo-Li Ming[d], Hongjun Song[d], Juan Song[a, b, 2]

Corresponding author: Juan Song
Email: juansong@email.unc.edu

This PDF file includes:

- Supporting materials and methods
- SI References
- Figures S1 to S13
- Legends for Datasets S1 and S2

Materials and Methods

Stereotaxic Injections

Stereotaxic injections were performed on animals 6-8 weeks of age as described in (1). The following coordinates were used for injections: Dentate Gyrus A/P -2.00 mm, M/L +/- 1.5 mm, D/V -2.15 mm, Medial Septum A/P .75 mm, M/L 0.0 mm, D/V -3.50, Diagonal Band of Broca A/P .75 mm, M/L +/-.60 mm, D/V -5.40 mm. AAVs were injected to the DG, MS, or DB, with a flow rate of 75 nl/min for the DG or 100 nl/min for the MS/DB. After injection, the needle was left in the brain for a total of 5 minutes, to allow for the dispersal of AAV particles in the targeted brain regions.

For anterograde tracing, mice were injected with 100 nl AAV5-EF1a-DIO-mCherry and AAV5-DIO-eYFP (UNC Vector Core) in the DB or MS, then were sacrificed 3 weeks post injection for immunohistochemistry.

For optogenetic experiments, we implanted optical fibers in the DG at the following coordinates: A/P -2.00 mm, M/L +/- 1.5mm, D/V -1.75 mm. Optical fibers were implanted after viral injections, and a head cap was created using dental cement. AAV5-EF1a-DIO-hChR2 (H134R)-eYFP (UNC Vector Core), AAV5-EF1a-DIO-hChR2 (H134R)-mCherry, or AAV9-ChaT-hChR2(H134R)-eGFP (Cat#: PT-2313, Brain VTA, China) are for optogenetic activation, while AAV5-EF1a-DIO-eArch3.0-eYFP (UNC Vector Core) are for optogenetic inhibition. For experiments where we combined in vivo fiber photometry with optogenetics, mice were unilaterally injected with 250 nl of AAVDJ-CaMKII-GCaMP6f (UNC Vector Core) or AAV9-mDlx-GCaMP6f (Addgene, Plasmid #83899) or AAVPHPeB-gfaABC1D-cyto-GCaMP8s (Addgene, Plasmid #186052) in the DG (A/P -2.00 mm, M/L - 1.5 mm, D/V -2.15 mm) and 250nl AAV9-hSyn-FLEX-ChrimsonR-tdTomato (UNC Vector Core) in the DB (A/P .75 mm, M/L -.60 mm, D/V -5.40 mm). We implanted two optical fibers above the viral injection sites with 20-degree angle in order to facilitate the simultaneous optical stimulation and calcium recording, one is for fiber photometry recording (A/P -2.00 mm, M/L - 2.25 mm, D/V -1.95 mm) and another fiber is for optogenetic simulation (A/P -2.00 mm, M/L - 0.75 mm, D/V -1.85 mm).

Fiber photometry recording

Briefly, the system consisted of a 488 nm excitation laser, a fluorescence cube, and a spectrometer. The 488 nm laser beams first launched into the fluorescence cube, then launched into the optical fibers. The GCaMP emission fluorescence collected from the fiber probe travelled back to the spectrometer. Only animals with strong GCaMP expression were included in the study. Spectral data was acquired by OceanView software (Ocean Optics, Inc) at 10 Hz and was synchronized to a 20 Hz video recording system to acquire the animal behavior.

Chemogenetic manipulation

For neurogenesis experiments, mice were injected with 200 nl AAV5-hSyn-DIO-hM3Dq-mCherry or AAV5-hSyn-DIO-mCherry (UNC Vector Core) in the DB bilaterally, and then after three weeks were administered CNO via drinking water at the concentration of 2.5 mg/ 200 ml for a total of 3 days. Fresh water bottles containing CNO-infused drinking water were replaced daily. For the morphology experiments using ChAT-Cre::Nestin-GFP mice, we chemogenetically inhibit DG GCs expressing AAV8-CaMKII-hM4Di-mCherry (Addgene, Plasmid #50477) via intraperitoneal injection of CNO at 1mg/kg (two injections total, every 4 hours) along with optogenetic activation of DB-DG cholinergic projections using the similar paradigms described above.

Edu Labeling

Proliferating cells were labeled using a thymidine analog EdU similar to that described in (1). Briefly, animals were intraperitoneally injected with EdU (4 mg/kg, 4 injections with 2-hour interval

for most neurogenesis studies, except for optogenetic inhibition with only 1 injection) on the last day of experiments. EdU was then visualized by using click chemistry reaction.

Tamoxifen administration

Tamoxifen was intraperitoneally injected (once daily at 80mg/kg for two days) to induce recombination in Gli1-CreER::Ai9 mice. Recombination without tamoxifen induction revealed no Ai9/tdTomato expression in the DG (SI Appendix, Fig. S13).

Image acquisition

For anterograde tracing experiments the entire DG was imaged using a 20x objective with 2x zoom, 1µm step, and 8x averaging using a resonant scanner. When quantifying adult neural stem cells, entire DGs were scanned with a Galvano scanner instead using a 20x objective with 2x zoom and 4x averaging and 1 µm step. For morphology analysis, images were acquired with a 60x objective, 8x averaging, 2x zoom, and 0.5 µm step.

Imaging analysis

For anterograde tracing, we utilized Imaris to perform a volumetric analysis of the projections from MS or DB. ROIs were manually drawn using hippocampal landmarks to distinguish between different regions. The reported values are a measurement of the sum of the volume of all objects within each ROI divided by ROI volume to normalize for different size ROIs. Hippocampal sections spanned from the dorsal DG to the start of the ventral DG.

For viral diffusion along the rostrocaudal axis of the DB and MS, three rostrocaudal levels per animal were quantified (+1.10 mm, +0.86 mm, and +0.50 mm). Please see viral labeling around the injecting sites (SI Appendix, Fig. S12).

For neurogenesis experiments, images were manually quantified using FIJI plugin under the Analyze/Cell Counter/Cell Counter tab. A total of 5 sections were quantified per animal spanning the dorso-ventral axis. For details, please refer to (2).

For morphology experiments, Image J was used to measure rNSC length from the center of the cell soma to the longest radial process. For bushy head analysis, Imaris was used to create ROIs around individual cells that were separated enough from other rNSCs.

Slice Electrophysiology

At 4 to 5 weeks after AAV5-EF1a-DIO-hChR2(H134R)-eYFP injections, ChAT-Cre::Nestin-GFP mice were anesthetized with isoflurane (5% in O_2) and transcardially perfused with ice-cold aCSF (N-methyl-D-glucamine, NMDG-based solution) containing the following (in mM): 92 NMDG, 30 NaHCO3, 25 glucose, 20 HEPES, 10 MgSO4, 5 sodium ascorbate, 3 sodium pyruvate, 2.5 KCl, 2 thiourea, 1.25 NaH2PO4, and 0.5 CaCl2, equilibrated with 95% O_2 and 5% CO_2 (pH 7.3, 305-315 mOsm). Brains were rapidly removed, and acute coronal slices (280 µm) containing the basal forebrain or hippocampus were cut using a Leica vibratome (VT1200, Germany). Next, slices were warmed to 34.5°C for 8 minutes. Then, slices were maintained in the holding chamber containing HEPES aCSF (in mM): 92 NaCl, 30 NaHCO3, 25 glucose, 20 HEPES, 5 sodium ascorbate, 3 sodium pyruvate, 2.5 KCl, 2 thiourea, 2 MgSO4, 2 CaCl2, 1.25 NaH2PO4 (pH 7.3, 305-315 mOsm) at room temperature for at least 1 hour before recording.

Individual slices were visualized under an upright microscope with differential interference contrast (IR-DIC) video microscopy and an IR-sensitive CCD camera (Scientific, FWCAM, USA). Responses were evoked by 5-ms light flashes (473 nm) delivered through a 40X objective attached to a microscope using an LED (Thorlabs, Canada). Patch pipettes with a resistance of 4–6 MΩ were pulled from borosilicate glass capillaries (1.5 mm outer diameter, 0.86 mm internal diameter, World Precision Instruments, USA) using a micropipette puller (PC-10, Narishige,

Japan). The internal solution used contained the following (in mM): 130 K-gluconate, 20 HEPES, 4 MgCl2, 4 Na-ATP, 2 NaCl, 0.5 EGTA, 0.4 Na-GTP (pH 7.2, 290 mOsm).

For circuit mapping experiments, we recorded from nestin-GFP+ cells in the dentate gyrus of the hippocampus, we recorded light-evoked currents under the voltage-clamp recordings (holding at -65 mV) by applying 8 Hz light pulses for 2 s at every 30 s. When needed, 100 µM d-(−)-2-amino-5-phosphonopentanoic acid (d-APV), 20 µM 6-cyano-7-nitroquinoxaline-2,3-dione (CNQX), and 10 µM atropine were added to block NMDA, AMPA/kainate and muscarinic ACh receptors, respectively. Light-evoked postsynaptic current amplitude was calculated at the peak of the first response after light pulses.

Recordings were conducted in the whole-cell configuration using a Multiclamp 700B amplifier (Axon Instruments, USA). Signals were filtered at 1 kHz and sampled at 10 kHz using the Digidata 1440A (Axon Instruments, USA), data acquisition was performed using pClamp 10.3 (Axon Instruments, USA). Series resistance (Rs) was monitored throughout all experiments and cells with Rs changes over 20% were discarded.

Split-Seq Library Preparation and Sequencing

Single-nucleus RNA sequencing was performed following the SPLiT-seq method with minor modifications (3). Nuclei isolated from flash-frozen DGs were performed as previously described (4, 5). Briefly, tissue was thawed, minced, and homogenized using 1 mL HB buffer (1 mM DTT, 0.15 mM spermine, 0.5 mM spermidine, EDTA-free protease inhibitor, 0.3% IGEPAL-630, 0.25 M sucrose, 25 mM MgCl2, 20 mM Tricine-KOH) for 5 to 10 strokes, then filtered through a 40 mm strainer, under layered with a cushion buffer (0.5 mM $MgCl_2$, 0.5 mM DTT, EDTA-free protease inhibitor, 0.88 M sucrose) to prevent damage to nuclei, and centrifuged at 2800 g for 10 minutes in a swinging bucket centrifuge at 4°C. The pellets were resuspended in 1 mL of cold PBS-RI (1x PBS + 0.05 U/ml RNase Inhibitor). The nuclei were passed through a 40 um strainer. 3 mL of cold 1.33% formaldehyde solution was then added to 1 mL of cells. Nuclei were fixed for 10 mins before adding 160 uL of 5% Triton X-100. We then permeabilized nuclei for 3 mins and centrifuged at 500 g for 3 mins at 4°C. Nuclei were resuspended in 500 uL of PBS-RI before adding 500 uL of cold 100 mM Tris-HCl pH 8. Then, nuclei were spun down at 500 g for 3 mins at 4°C and resuspended in 300 uL of cold 0.5 X PBS-RI. Finally, nuclei were again passed through a 40 mm strainer and then counted on a hemocytometer, diluted to 1,000,000 cells/mL. mRNA from single nuclei were tagged 3 rounds with barcoded primers, with in-cell ligations using T4 DNA ligase within 96-well plates. Plates were incubated for 30 mins at 37°C with gentle shaking (50 rpm) to allow hybridization and ligation to occur. The ligation products were purified with Dynabeads MyOne Streptavidin C1 beads. After washing beads once with 10 mM Tris and 0.1% Tween-20 solution and once with water, beads were resuspended into a solution containing 110 uL of 2X Kapa HiFi HotStart Master Mix, 8.8 uL of 10 mM stocks of primers BC_0062 and BC_0108, and 92.4 uL of water. PCR thermocycling was performed as follows: 95°C for 3 mins, then five cycles at 98°C for 20 s, 65°C for 45 s, 72°C for 3 mins. After these five cycles, Dynabeads beads were removed from the PCR solution and EvaGreen dye was added at a 1X concentration. Samples were again placed in a qPCR machine with the following thermocycling conditions: 95°C for 3 mins, cycling at 98°C for 20 s, 65°C for 20 s, and then 72°C for 3 mins, followed by a single 5 mins at 72°C after cycling. Once the qPCR signal began to plateau, reactions were removed.

PCR reactions were purified using a 0.8X ratio of KAPA Pure Beads and cDNA concentration was measured using a qubit. For tagmentation, a Nextera XT Library Prep Kit was used. 600 pg of purified cDNA was diluted in water to a total volume of 5 uL.10 uL of Nextera TD buffer and 5 uL of Amplicon Tagment enzyme were added to bring the total volume to 20 uL. After mixing by pipetting, the solution was incubated at 55°C for 5 mins. A volume of 5 uL of neutralization buffer was added and the solution was mixed before incubation at room temperature for another 5 mins.

PCR was then performed with the following cycling conditions: 95°C for 30 s, followed by 12 cycles of 95°C for 10 s, 55°C for 30 s, 72°C for 30 s, and 72°C for 5 mins after the 12 cycles. 40 uL of this PCR reaction was removed and purified with a 0.7X ratio of SPRI beads to generate an Illumina-compatible sequencing library.

Single Nuclei RNA-seq data analysis

FASTQ files were deconvoluted and transcript abundance was estimated using zUMIs v2.9.6 (6) and GENCODE vM26 annotations (7). Bases 1-66 were extracted from the R1 file, corresponding to cDNA sequence, and for the R2 file, bases 1-10 represented UMI, and bases 11-18, 49-56, and 87-94 represented the three barcodes. Internally, zUMIs performs a two-pass alignment using STAR v2.7.3a (8) with the additional parameter "--limitSjdbInsertNsj 2000000". Barcodes and UMIs were also filtered for quality using "num_bases=1, phred=10". We then generated cell x genes matrices. Data were then imported into R v3.6 and Seurat V3 (9) objects were created for each individual sample before being merged. Cells were filtered such that there were at least 1000 UMIs and 500 genes detected, and fewer than 10% of the transcripts were mitochondrially contributed. Data were then scaled and normalized using scTransform (10) and integrated using Seurat. Clusters were identified using Louvain-Jaccard clustering with multilevel refinement (resolution=2) performed on the top 100 PCs. Marker genes of each cluster were identified using the FindMarkers function in Seurat, and differential expression analysis was performed using RNA CPMs in R using Wilcoxon Rank-Sum test. Differentially expressed genes were considered significant with a nominal p-value < 0.01. We determined whether DB cholinergic afferent activation regulated DG cells by intersecting our data with gene ontology databases using g:Profiler2 (11) and looking at GO terms associated with Biological Processes (BP) only.

SI References

1. L. J. Quintanilla, C. Y. Yeh, H. Bao, C. Catavero, J. Song, Assaying Circuit Specific Regulation of Adult Hippocampal Neural Precursor Cells. *J Vis Exp* 10.3791/59237 (2019).
2. Y. D. Li *et al.*, Activation of hypothalamic-enhanced adult-born neurons restores cognitive and affective function in Alzheimer's disease. *Cell Stem Cell* **30**, 415-432 e416 (2023).
3. A. B. Rosenberg *et al.*, Single-cell profiling of the developing mouse brain and spinal cord with split-pool barcoding. *Science* **360**, 176-182 (2018).
4. Y. Su *et al.*, Neuronal activity modifies the chromatin accessibility landscape in the adult brain. *Nat Neurosci* **20**, 476-483 (2017).
5. X. Qian *et al.*, Sliced Human Cortical Organoids for Modeling Distinct Cortical Layer Formation. *Cell Stem Cell* **26**, 766-781 e769 (2020).
6. S. Parekh, C. Ziegenhain, B. Vieth, W. Enard, I. Hellmann, zUMIs - A fast and flexible pipeline to process RNA sequencing data with UMIs. *Gigascience* **7** (2018).
7. J. Harrow *et al.*, GENCODE: the reference human genome annotation for The ENCODE Project. *Genome Res* **22**, 1760-1774 (2012).
8. A. Dobin *et al.*, STAR: ultrafast universal RNA-seq aligner. *Bioinformatics* **29**, 15-21 (2013).
9. T. Stuart *et al.*, Comprehensive Integration of Single-Cell Data. *Cell* **177**, 1888-1902 e1821 (2019).
10. C. Hafemeister, R. Satija, Normalization and variance stabilization of single-cell RNA-seq data using regularized negative binomial regression. *Genome Biol* **20**, 296 (2019).

11. L. Kolberg, U. Raudvere, I. Kuzmin, J. Vilo, H. Peterson, gprofiler2 -- an R package for gene list functional enrichment analysis and namespace conversion toolset g:Profiler. *F1000Res* **9** (2020).

Supplementary Figures

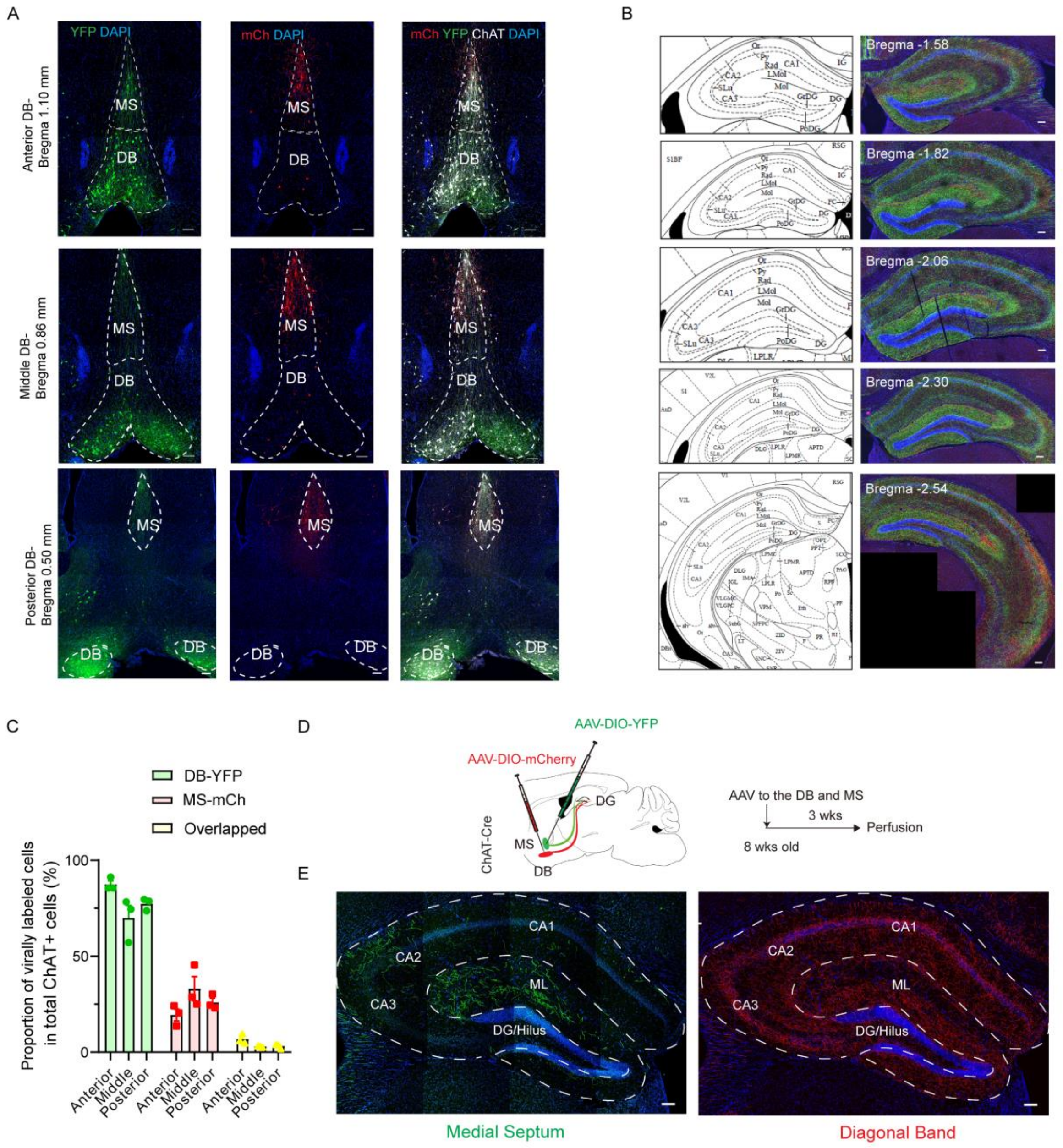

Fig. S1. Distinct patterns of cholinergic projections from Medial Septum and Diagonal Band of Broca to DG.

(A) Confocal images showing the different levels of viral expression in the DB or MSco-stained with ChAT antibody in virus injection experiments of present studies. Scale bar = 100 µm.

(B) Confocal images of hippocampal sections used to quantify volumetric projections spanning about 2 mm of the hippocampus. Scale bar = 100 µm

(C) The proportion of viral and ChAT co-expressed neurons in total ChAT positive neurons of different levels of the DB. n=3 animals, Bars indicate mean +/- S.E.M.

(D) Schematic diagram of AAVs targeting MS and DB using dual fluorophores (mCherry for the DB, and YFP for the MS).

(E) Confical images of hippocampus with ROIs drawn around respective hippocampal subregions. Scale bar 100 µm.

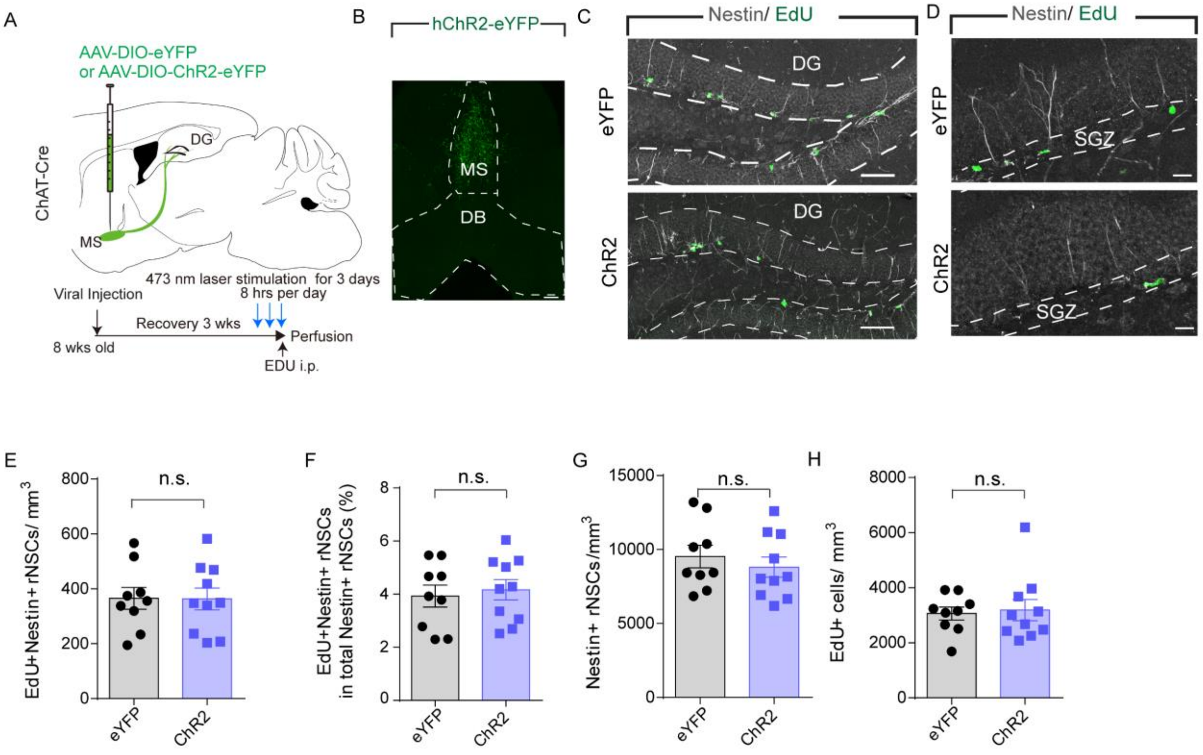

Fig. S2. Stimulation of MS-DG cholinergic projections has no effects on rNSC proliferation.

(A) Schematic diagram of timeline and experimental setup for optogenetic stimulation of MS cholinergic afferents and rNSC proliferation analysis.

(B) Confocal image of MS targeted AAV viral injection and ChR2 afferents in the DG. DB scale bar 100 DG Scale bar 20 µm.

(C) Confocal image of DG used to quantify rNSC proliferation. Scale bar = 100 µm

(D) Confocal image of proliferating rNSCs with colocalization of Nestin and EdU (arrow heads). Scale bar = 10 µm

(E) Density of proliferating rNSCs. eYFP: n = 9 animals; ChR2: n = 10 animals.

(F) Percent of proliferating rNSCs. eYFP: n = 9 animals; ChR2: n = 10 animals.

(G) Density of rNSCs. eYFP: n = 9 animals; ChR2: n = 10 animals.

(H) Density of overall proliferating progeny. eYFP: n = 9 animals; ChR2: n = 10 animals.

Data were presented as mean +/- S.E.M.

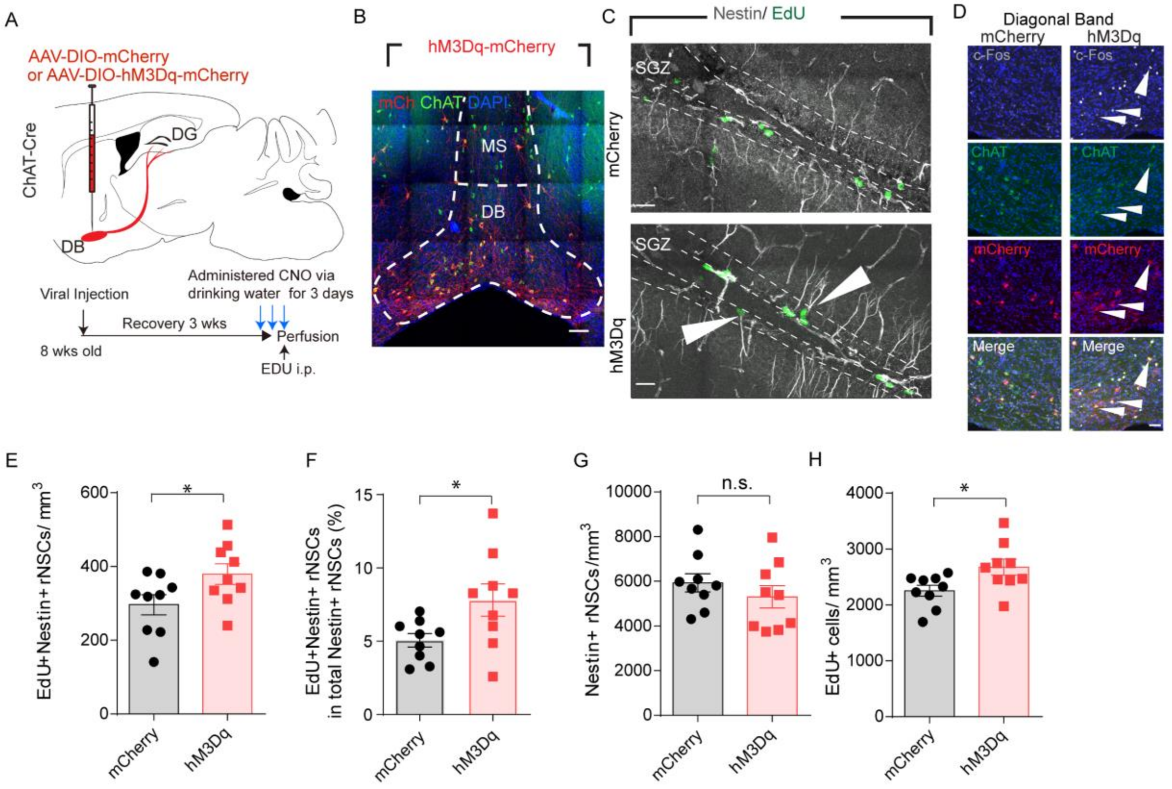

Fig. S3. Chemogenetic activation of DB neurons increases proliferation of rNSCs.

(A) Schematic diagram of timeline and experimental setup for chemogenetic stimulation of DB cholinergic afferents and rNSC proliferation analysis.

(B) Confocal image of DB targeted by AAVs and DB mCh cells stained with ChAT. Sscale bar =100µm,

(C) Confocal image of proliferating rNSCs with colocalization of Nestin and EdU (arrow heads). Scale bar = 50 µm.

(D) Confocal image of chemogenetically activated DB cells stained with ChAT, mCherry, and c-Fos antibodies. Scale bar = 50 µm

(E) Density of proliferating rNSCs. mCherry: n = 9 animals; hM3Dq: n = 9 animals. $P = 0.0497$ by student's *t*-test .

(F) Percent of proliferating rNSCs. mCherry: n = 9 animals; hM3Dq: n = 9 animals. $P = 0.0348$ by student's *t*-test.

(G) Density of rNSC pool. mCherry: n = 9 animals; hM3Dq: n = 9 animals.

(H) Density of overall proliferation. mCherry: n = 9 animals; hM3Dq: n = 9 animals. $P = 0.0266$ by student's *t*-test.

Data were presented as mean +/- S.E.M.

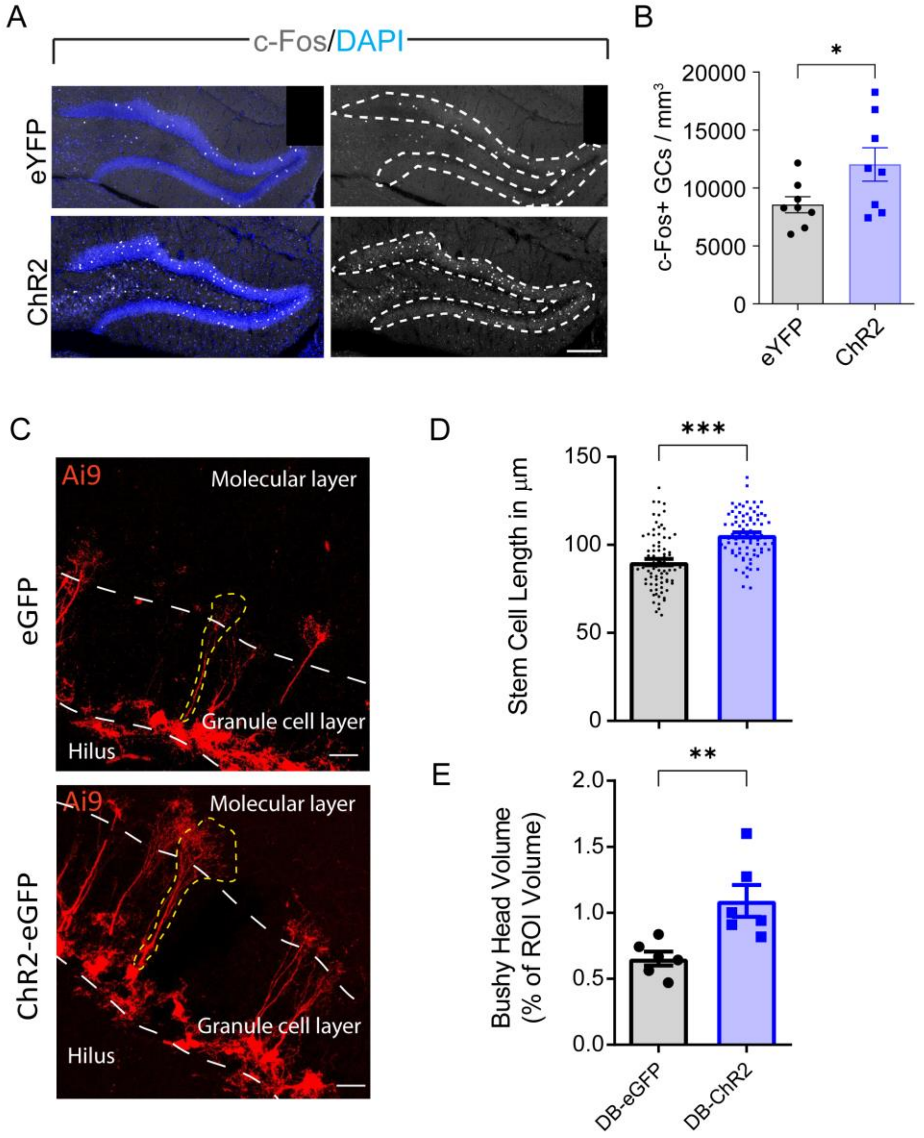

Fig. S4. DB-DG cholinergic circuit activation increases c-Fos expression in granule cells .and alters rNSC morphogenesis in Gli-CreER::Ai9 mice.

(A) Representative confocal images of DG sections quantified for c-Fos expression after DB optogenetic stimulation in ChAT-Cre mice. Scale bar = 100 µm.

(B) Quantification of c-Fos in Granule cells, bars indicate mean +/- S.E.M. eYFP: n = 8 animals; ChR2: n = 8 animals. P = 0.0348 student's t-test.

(C) Confocal images of rNSC bushy heads from a Gli1-CreER :: Ai9 animal upon sham or optogenetic activation of DB-DG cholinergic terminals. Scale bar = 20 µm.

(D) Mean length of rNSCs in eGFP and ChR2-eGFP groups. eYFP: n = 80 cells from 3 animals; ChR2: n = 80 cells from 3 animals. P <0.0001.

(E) Volume of rNSC bushy heads in eGFP and ChR2-eGFP groups. eGFP: n = 6 slices from 3 animals; ChR2: n = 6 slices from 3 animals., P = 0.0075.

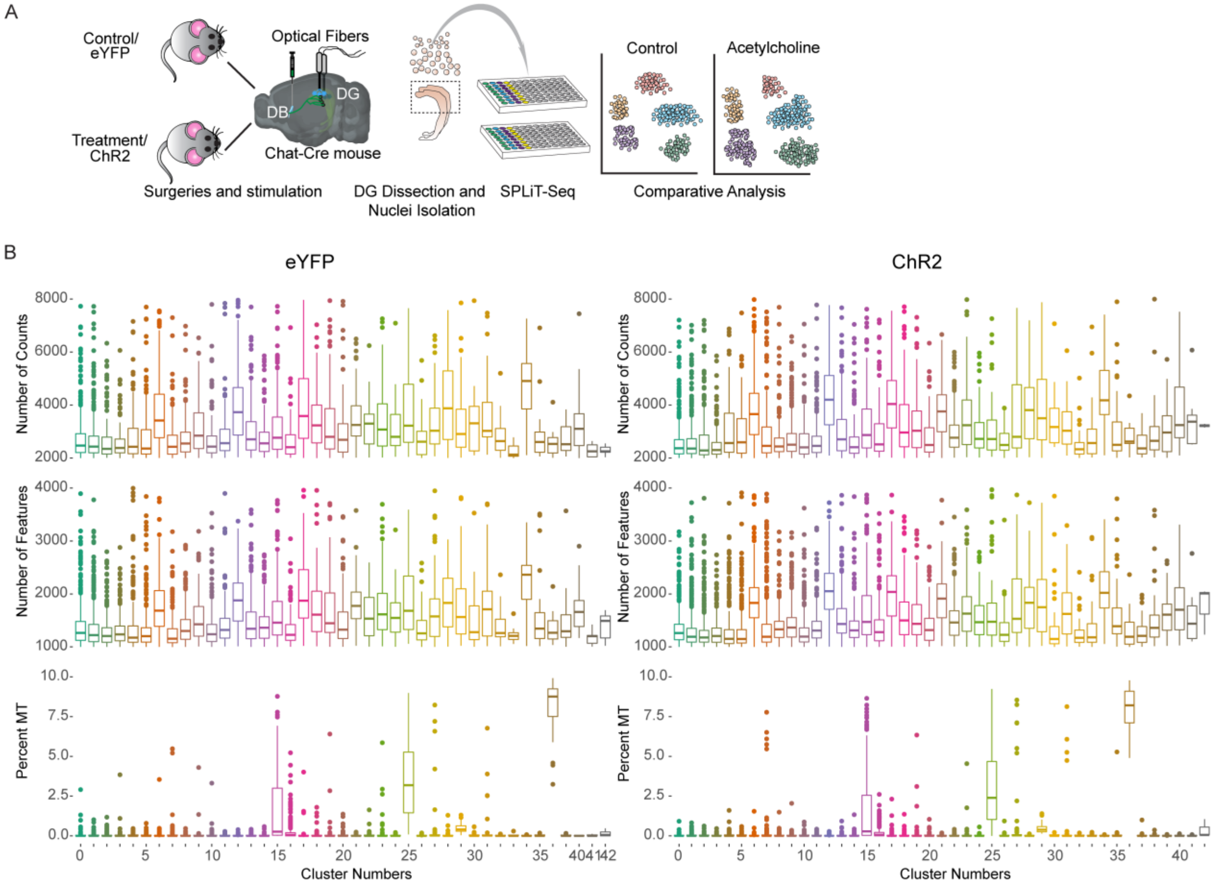

C

Cluster Number	0	1	2	3	4	5	6	7	8	9	10	11	12	13	14	15	16	17	18	19	20
eYFP	881	788	581	643	605	547	517	519	536	112	453	399	295	454	235	295	225	194	121	154	156
ChR2	877	800	694	632	559	562	546	512	489	904	397	400	462	218	370	277	280	302	209	167	165

Cluster Number	21	22	23	24	25	26	27	28	29	30	31	32	33	34	35	36	37	38	39	40	41	42
eYFP	123	102	127	121	107	94	104	95	97	81	59	54	19	31	44	39	28	0	0	16	11	10
ChR2	192	189	158	131	116	108	97	76	66	68	74	49	83	67	54	31	38	65	54	21	13	3

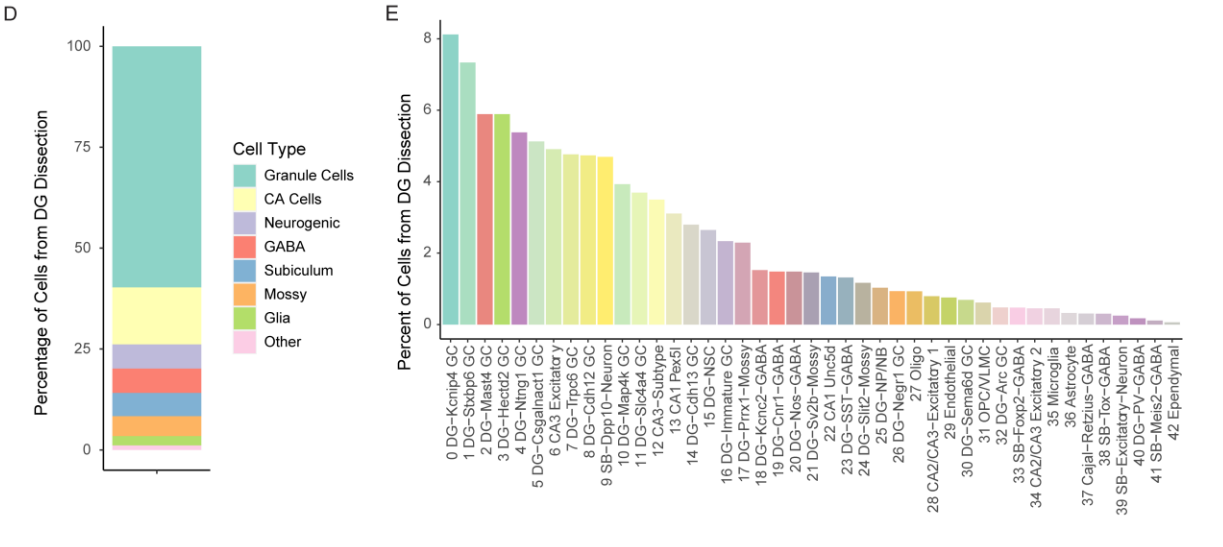

Fig. S5. Quality control of snRNA-seq clusters.

(A) Experimental schematic for snRNA-seq experiment upon circuit-specific stimulation of DB cholinergic afferents. Dorsal DGs were microdissected from a total of 10 animals (5 animals for eYFP control, 5 animals for ChR2, 10 weeks old)

(B) Boxplots showing upper quartile, median, and lower quartile of the total number of counts (UMIs), number of features (genes), or percent of mitochondrial reads for each cluster separated by eYFP and ChR2. Cells that did not meet the minimum requirements for any of these 3 criteria were excluded.

(C) Total number of cells in each cluster for both Control and ChR2 that passed quality control metrics.

(D) Percentage of cell classes for all clusters dissected from the DG.

(E) Percentage of cells within each cluster from all dissected cells from our experiment.

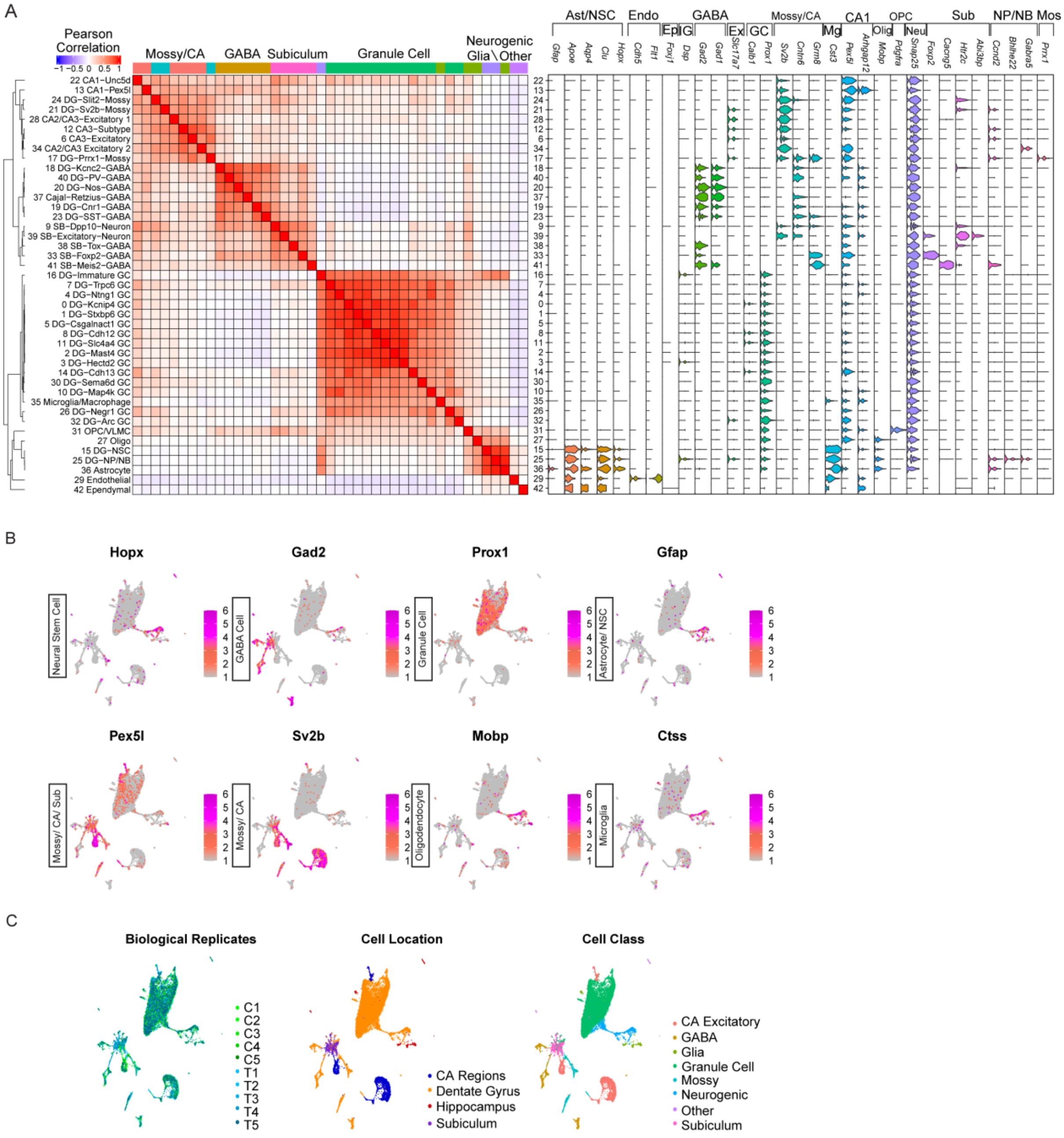

Fig. S6. Establishment of cluster identities using marker genes.

(A) Pearson pairwise correlation plot using the mean expression of the top fifty most representative genes for each cluster and a violin plot showing expression of marker genes within each cluster. Violin plot has labels for each cluster above marker genes that correspond to the following: Ast/NSC - Astrocyte/Neural Stem Cell, Endo – Endothelial, Ep – Ependymal, IG – Immature Granule Cell, GABA – GABA interneuron, Ex – Excitatory Neuron, GC – Granule cell, Mossy/CA – Mossy cells/ Cornu ammonis, Mg – Microglia, CA1 – Cornu ammonis 1, Olg – Oligodendrocyte, OPC - Oligodendrocyte Progenitor Cell, Neu – Neuron, Sub -Subiculum, NP/NB – Neural Progenitor/Neuroblast, Mos – Mossy Cells.

(B) UMAP plots showing expression of different cell-type-specific markers. Plot title shows labeled marker gene and legend shows expression.

(C) Dimension reduction of clusters using UMAP plots to show clusters separated by biological replicates, cell location, or cell class.

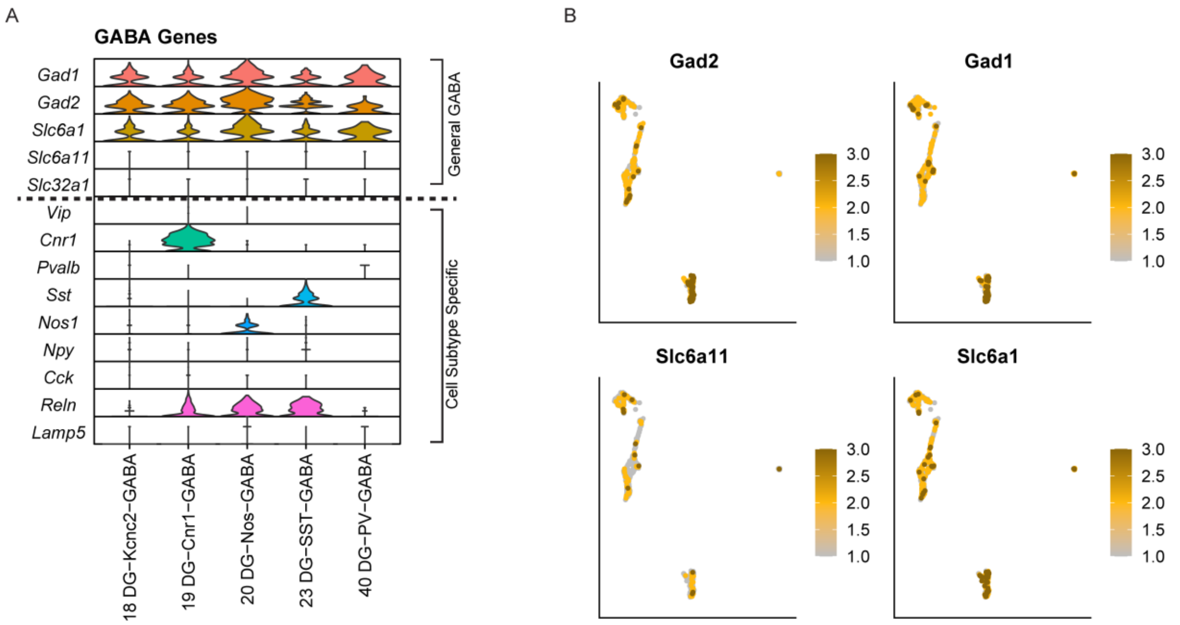

Fig. S7. Identification of GABA interneuron subclasses.

(A) Violin plot showing major GABA markers that are both shared and exclusive to certain cell types in clusters. Marker genes below indicate cell-type-specific markers.

(B) UMAP plots showing expression of 4 general GABA markers in DG-GABA clusters subset from the original clusters.

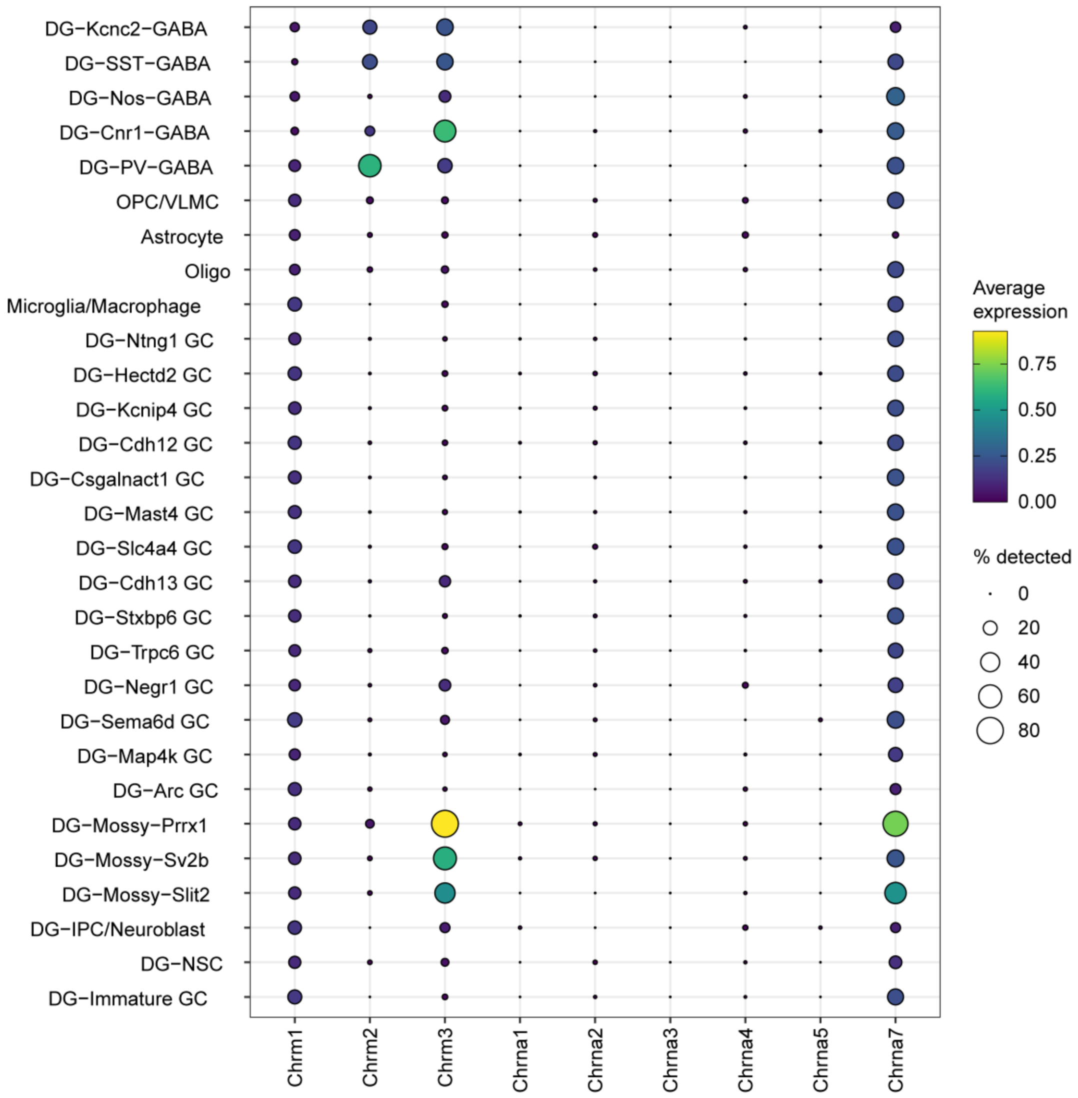

Fig. S8. Expression of cholinergic receptors in DG clusters. Dot plot showing SCT normalized expression of cholinergic receptor genes in DG clusters from snRNA-seq analysis. Each cluster name and class are indicated in the names on the left and genes are ordered by nicotinic receptors and muscarinic receptors. If receptor genes are not present, they were not detected in our SCT normalized expression.

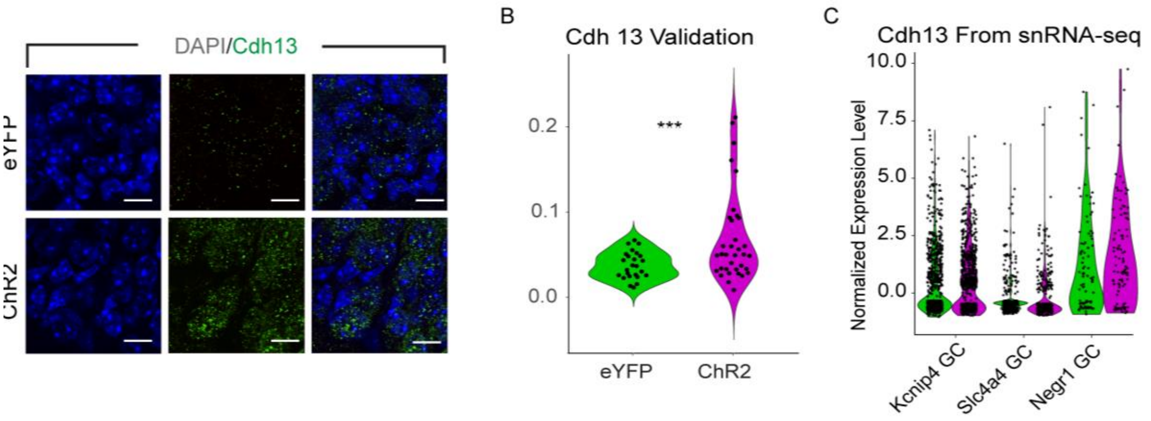

Fig. S9. Validation of Cdh13 expression in GCs.

(A) Representative confocal image of Cdh13 Immunofluorescence. By note, consistent with the sn-RNA-Seq data, we only detected a subset of GCs expressing Chd13 that is upregulated after cholinergic circuit stimulation. Scale bar = 20 µm

(B) Violin plot shows sum of Cdh13 expression volumes within each cell using Imaris. n = (25,35) cells from 3 biological replicates per condition. *P* = 0.0023 student's *t*-test and clusters which have differentially expressed levels of Cdh13.

(C) Violin plot shows normalized expression of Cdh13 in the snRNAseq experiment in three GC clusters showing the ChR2 group has higher expression.

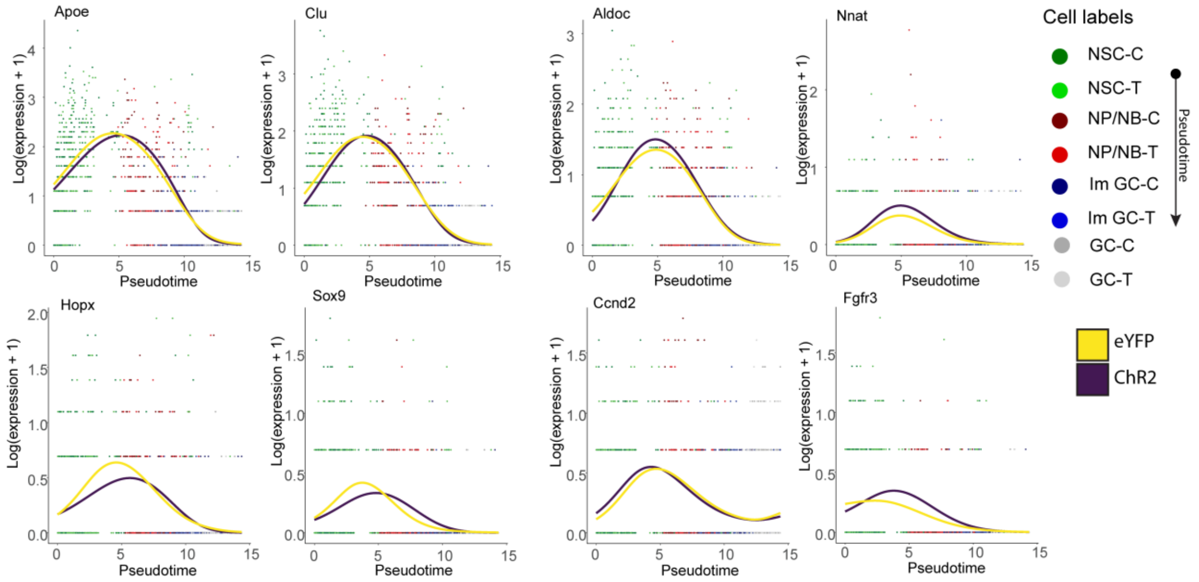

Fig. S10. Pseudotime analysis of neurogenic lineage. Pseudotime representation of neurogenic clusters generated using Slingshot. Pseudotime goes from left to right. eYFP (control) clusters are darker and ChR2 (treatment) clusters are lighter.

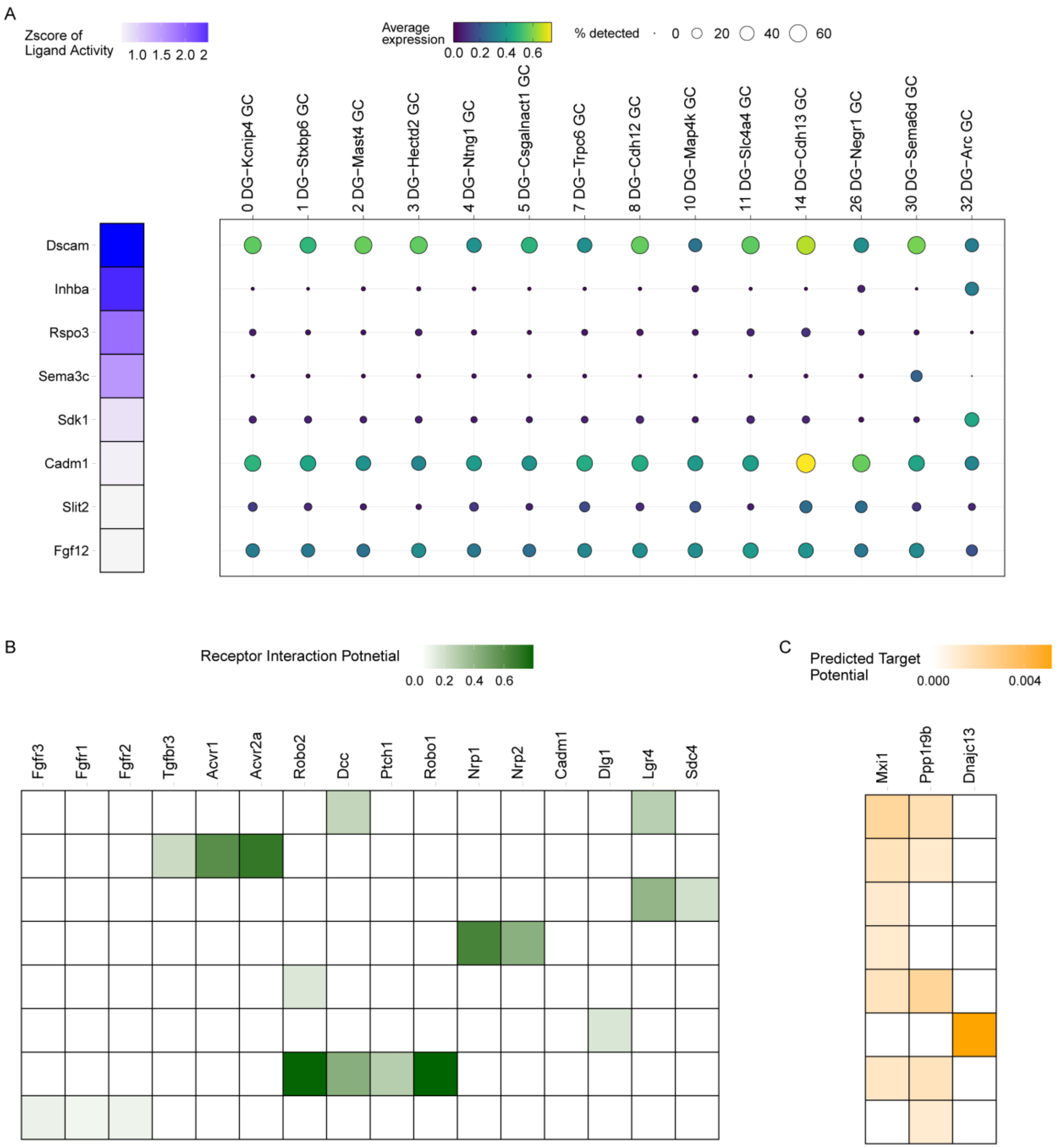

Fig. S11. NicheNet analysis reveals secretory ligands from GCs for rNSC regulation.

(A) NicheNet predicted ligands ordered by Z-Score taken from Pearson's Correlation and dot plot showing expression of ligands in sender clusters (GCs) used to generate predictions about receptor and target gene interactions.

(B) Predicted ligand receptors within rNSCs from snRNA expression data using Nichenet.

(C) Predicted target genes within rNSCs from snRNA expression data using Nichenet.

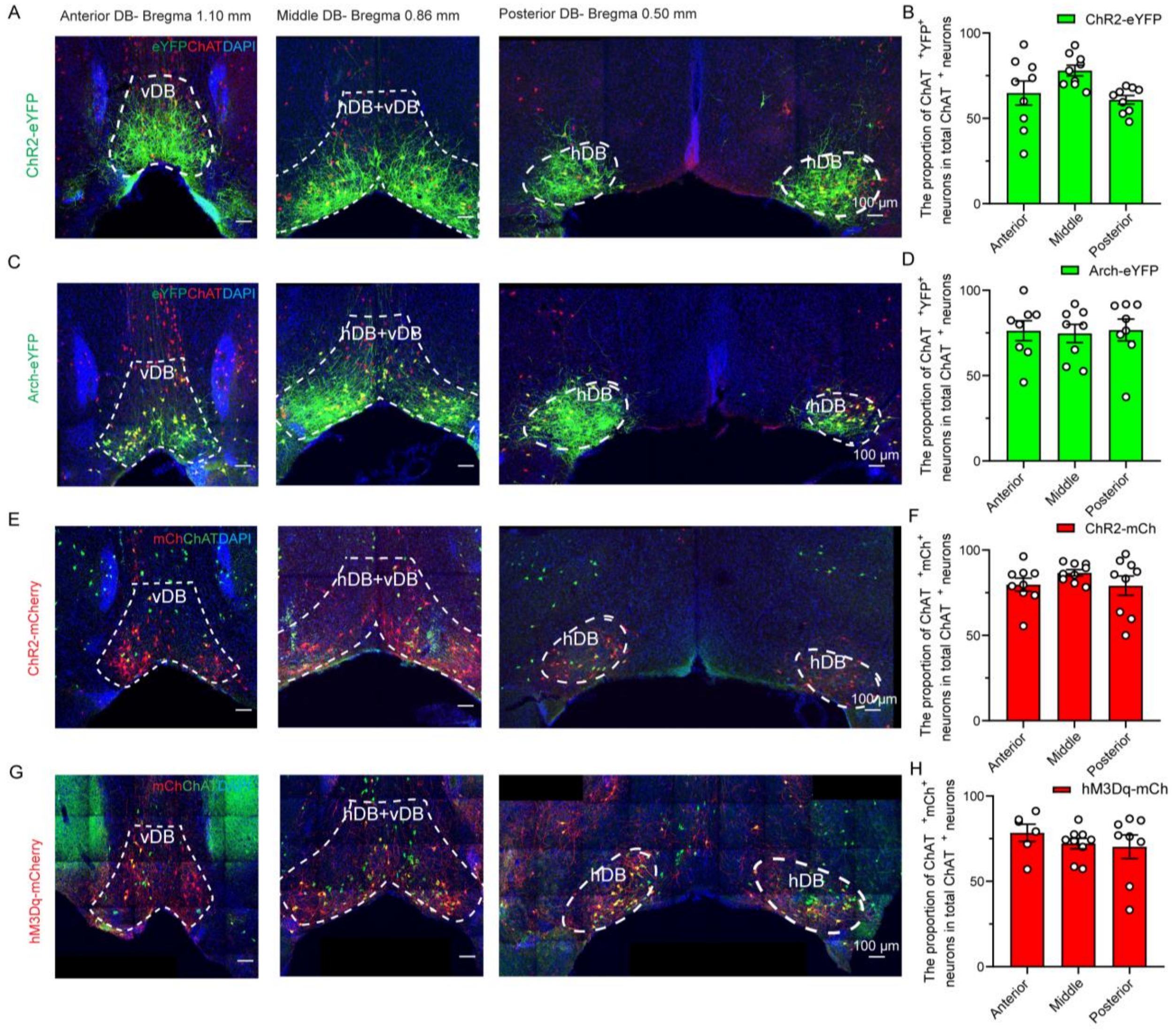

Fig. S12. Identification of viral expression in the DB.

(A, C, E, G) Confocal images showing the different levels of viral expression in the DB co-stained with ChAT antibody in virus injection experiments of present studies. The coronal section of anterior DB is selected as Bregma = 1.10, the coronal section of middle DB is selected as Bregma = 0.86, and the coronal section of posterior DB is selected as Bregma = 0.50. vDB, the vertical limb of the diagonal band; hDB, the horizontal limb of the diagonal band. Scale bar = 100 µm.

(B, D, F, H) The proportion of viral and ChAT co-expressed neurons in total ChAT positive neurons of different levels of the DB. B, n = 9 animals (Fig. 2A-H); D, n = 8 animals (Fig. 2I-P); E, n = 9 animals (Fig. 6E-H); H, n = 9 animals (SI Appendix, Fig. S3). Bars indicate mean +/- S.E.M.

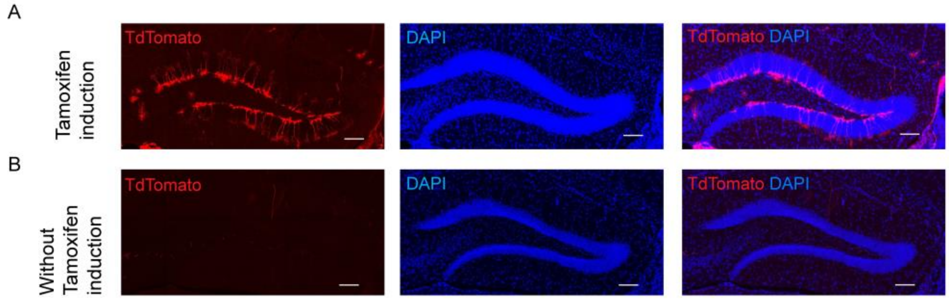

Fig. S13. Validating the specificity of the Gli1-CreER::Ai9 mice in labeling neurogenic lineage upon Tamoxifen induction.

(A) Confocal images showing prominent signals of Ai9 expression in the DG subgranular zone (SGZ) of the Gli1-CreER:: Ai9 mice 7 days post tamoxifen induction.

(B) Confocal images showing no signals of Ai9 expression in the SGZ of the Gli1-CreER :: Ai9 mice in the absence of tamoxifen induction.

Dataset S1. Cell-type specific DEGs in response to DB-DG cholinergic circuit stimulation.

Dataset S2. Cell-type specific GO terms in response to DB-DG cholinergic circuit stimulation.
